# ﻿Asymmetric mitonuclear interactions trigger transgressive inheritance and mitochondria-dependent heterosis in hybrids of the model system *Pleurotus ostreatus*

**DOI:** 10.3897/imafungus.16.165520

**Published:** 2025-10-31

**Authors:** Edurne Garde, Gumer Pérez, Idoia Jiménez, María Isabel Calvo, Antonio G. Pisabarro, Lucía Ramírez

**Affiliations:** 1 Institute for Multidisciplinary Research in Applied Biology (IMAB), Public University of Navarre (UPNA), Pamplona, Navarre, Spain Public University of Navarre (UPNA) Pamplona Spain; 2 Department of Pharmaceutical Sciences, School of Pharmacy and Nutrition, University of Navarra, Irunlarrea 1, 31008 Pamplona, Navarre, Spain University of Navarra Pamplona Spain; 3 IDISNA—Instituto de Investigación Biosanitaria de Navarra, 31008 Pamplona, Navarre, Spain E.G. and G.P. have equally contributed IDISNA—Instituto de Investigación Biosanitaria de Navarra Pamplona Spain

**Keywords:** Electron transport chain, hybrid breakdown, mitochondrial dysfunction, mitonuclear incompatibility, oxidative stress

## Abstract

Mitonuclear interactions are crucial in governing mitochondrial function, development and responses to stress in eukaryotic organisms. In this study, we explored how varying mitochondrial haplotypes affect the phenotype and oxidative stress response using hybrids of the basidiomycete *Pleurotus ostreatus* (*P. ostreatus*) as a model system. By performing reciprocal crosses between monokaryotic strains with distinct nuclear and mitochondrial genomes, we identified notable differences in growth rates, accumulation of reactive oxygen species (ROS) and gene expression patterns. Hybrids with incompatible mitonuclear combinations displayed slower growth and elevated expression of genes — some showing transgressive inheritance — associated with the Electron Transport Chain (ETC) and antioxidant defences. Mitochondria-dependent heterosis was observed in hybrids sharing the same nuclear background, but differing in mitochondrial genome, suggesting that mitonuclear incompatibilities can result in oxidative imbalance and compromised fungal performance. This experimental approach opens wide possibilities for exploring mitonuclear interactions and highlights the significance of mitonuclear co-adaptation in an edible mushroom, offering valuable insights for enhancing hybrid breeding programmes by accounting for the role of mitonuclear interactions in shaping quantitative traits related to mushroom yield.

## ﻿Introduction

In eukaryotic organisms, the nuclear and organellar genomes co-evolve through reciprocal genetic changes driven by natural selection, ensuring functional coordination. This co-evolution often involves mutual adjustments in the selective constraints of interacting proteins. Within isolated taxa, such coordination is shaped by intrinsic selection, which promotes compatibility between nuclear and organellar genomes to maintain optimal molecular function. In contrast, extrinsic selection favours organelle traits that enhance fitness in specific environmental contexts (Burton et al. 2013).

When individuals from genetically distinct populations interbreed, their offspring inherits a combination of nuclear and organellar genomes that have not co-evolved. This genomic recombination can disrupt previously fine-tuned genetic interactions, resulting in novel allele combinations that have not undergone joint evolutionary refinement (Bateson 1909; Dobzhansky 1937; Muller 1942). Such disruptions can lead to genetic incompatibilities that reduce hybrid fitness, a phenomenon known as “hybrid breakdown”or “outbreeding depression”(Ellison and Burton 2008a; Gershoni et al. 2009; Burton and Barreto 2012; Burton et al. 2013; Hill 2016; Sloan et al. 2017; Hill et al. 2019; Wang et al. 2024). This means that F_1_ traits can fall outside the parental range. This effect tends to be more pronounced in the F_2_ and later generations. Another possible F_1_ outcome is heterosis or hybrid vigour, a phenomenon that occurs when interspecific hybrids or intraspecific crossbred F_1_ exhibit improved fitness compared to both parental species or strains. This could be explained by two models. The first explanation suggests that enhanced growth in hybrids results from the masking of harmful mutations or from beneficial interactions between parental alleles. The second proposes that it arises from impaired systems that would normally limit their growth, thus making hybrid vigour a result of disrupted regulation rather than of enhanced promotion of growth (Bar-Zvi et al. 2017). Heterosis is attributed to genetic mechanisms such as dominance, overdominance and epistasis (Burton 1990b; Burton et al. 2006; Ellison and Burton 2008b; Meiklejohn et al. 2013; Shapira et al. 2014; Strom and Bushley 2016; Bar-Zvi et al. 2017; Herbst et al. 2017; Schneemann et al. 2022; Wang et al. 2022; McDiarmid et al. 2024; Wang et al. 2024).

Hybrid breakdown and heterosis have been documented in hybrids between divergent populations of *Tigriopus californicus*, a copepod native to the west coast of North America. Hybrid breakdown was reported as: reduced mitochondrial efficiency (Blier 2001; Ellison and Burton 2008a; Pichaud et al. 2013), decreased ATP production (Ellison and Burton 2006, 2008b; Healy and Burton 2020; Han and Barreto 2021), elevated oxidative DNA damage (Barreto and Burton 2013), upregulated stress-response pathways and transgressive gene expression (overdominance/underdominance) of misregulated genes, including those involved in the ETC (Barreto et al. 2015) and life-history traits such as fecundity, survivorship and developmental rate (Burton 1990a; Edmands 1999).

In budding yeast hybrids, it was demonstrated by transgressive gene expression evidenced by multiple signs of impaired regulation of growth-related pathways, such as loss of programmed cell-cycle delays, weakened repression of respiratory metabolism and altered slowdown of growth under stress, which were interpreted as dysregulation of safeguard mechanisms in hybrids (Herbst et al. 2017). Similar findings were reported in hybrids of catfish belonging to the genus *Ictalurus* (Wang et al. 2022).

Extensive research has addressed mitonuclear interactions in both *Ascomycota* and *Basidiomycota*. In *Ascomycota*, these interactions are strongly associated with ageing, mitochondrial DNA damage and oxidative stress and contribute to population divergence (Osiewacz 1995, 1997; Biot-Pelletier et al. 2023; Nguyen et al. 2023). Oxidative stress occurs when the generation of ROS exceeds the cell’s capacity to neutralise or detoxify them (Osiewacz and Kimpel 1999; Paliwal et al. 2014; Pizzino et al. 2017). In *Basidiomycota*, particularly in species of the *Heterobasidion* genus, mitonuclear interactions influence key traits such as pathogenicity, saprophytic ability and growth, highlighting the evolutionary and functional significance of mitonuclear epistasis in this group (Olson and Stenlid 2001; Giordano et al. 2018; Clergeot and Olson 2021). It was reported by De La Bastide et al. (De La Bastide et al. 1997) that, in the edible basidiomycete *Agaricus bisporus*, the mitochondrial type influences its growth rate and, more recently, Zhang et al. (Zhang et al. 2021) stated that the edible yellow chanterelles (*Cantharellus cibarius*) could be an excellent model system for studying the evolution of mitonuclear genome interactions.

*P. ostreatus* is one of the most widely cultivated and economically important edible mushroom globally, ranking after *Agaricus bisporus* and ahead of other notable species, including *Lentinus edodes*, *Pleurotus eryngii*, *Flammulina velutipes*, *Agaricus marmoreus*, *Cyclocybe cylindracea* and *Auricularia heimuer*. These fungi have been extensively studied through breeding programmes and molecular analyses (Nakazawa et al. 2024) and are established as valuable model systems for experimental research.

In the lignin-degrading, heterothallic strain *P. ostreatus* dN001 (n) (Division *Basidiomycota*) (Nakazawa et al. 2024), our group developed genetic and molecular markers, including those linked to mating type genes (Larraya et al. 1999a, 2000; Castanera et al. 2012, 2014; Alfaro et al. 2016) which enabled a straightforward identification of compatible monokaryons and the establishment of heterokaryotic mycelia capable of producing basidiospores under optimal conditions (Larraya et al. 2002, 2003). These markers also facilitated the construction of the first *P. ostreatus* genetic linkage map (Larraya et al. 2000), which localised qualitative traits — such as genes encoding hydrophobins, laccases, manganese peroxidases, copper transporters and telomeric sequences — as well as quantitative traits related to lignin-degrading gene expression (Peñas et al. 2005; Pérez et al. 2009; Castanera et al. 2012, 2013; Parenti et al. 2013) and mushroom production traits, including growth rate, yield, earliness, number of fruiting bodies, colour and fleshiness (Larraya et al. 2002, 2003).

Dedikaryotisation of dN001 (n) yielded two monokaryotic protoclones: the fast-growing PC9 and the slow-growing PC15 (Larraya et al. 1999b). Genome sequences (Grigoriev et al. 2014) revealed strong synteny, over 12,000 genes and numerous transposable elements (Castanera et al. 2016). Single nucleotide polymorphisms (SNPs) in both genomes enabled the determination of parental alleles and those of offspring (Castanera et al. 2016), facilitating: i) identification of strains with pronounced degeneration and ii) selection of strains for the experiments described herein.

Mushroom breeders have expressed concern over the decline in performance of commercial hybrid strains subjected to long-term subculturing as *P. ostreatus* strain dN001 (n) which has been used as a model organism in our laboratory since 1999 (Nakazawa et al. 2024). We hypothesise that fitness decline in F_1_, F_2_ and advanced generations may result from disrupted co-evolved mitonuclear interactions leading to hybrid breakdown, whereas heterosis or hybrid outperformance with transgressive inheritance observed in some hybrid strains may reflect gene compensation in new heterozygote hybrids or physiological dysregulation due to clashes between divergent genomes.

In this study, we will examine the growth rate under different temperatures and carbon sources, as well as the expression profile of ETC (*nd1*, *bcs1*, *rip1*, *cox4* and *cox5b*) and detoxification genes (*sod1*, *cat* and *gpx*) in parental and hybrid strains. The rationale underlying this selection was their upregulation in the slow-growing *P. ostreatus* strains which exhibited pronounced symptoms of degeneration (Pérez et al. 2021).

The mitochondrial gene *nd1*, which encodes a subunit of NADH dehydrogenase (ubiquinone), is one of seven mitochondrial-encoded genes (*nd1*–*nd6*, *nd4l*) that comprise Complex I of the ETC, essential for the mitochondrial respiratory function (Wirth et al. 2016; Lin et al. 2024). The *bcs1* and *rip1* are part of Complex III. The first one is an assembly factor essential for Complex III biogenesis, while *rip1* encodes a Rieske iron-sulphur protein critical for electron transfer and proton translocation (Ndi et al. 2018; Hou et al. 2023). The *cox4* gene encodes a protein that is part of the Complex IV, which catalyses the final step of the mitochondrial ETC and serves as a key regulatory site in oxidative phosphorylation. In contrast, *cox5b* is a nuclear gene encoding a Complex IV subunit that, in yeast, is expressed under hypoxic conditions, unlike *cox5a* which is expressed under normoxia (Burke et al. 1997; Castello et al. 2008; Liu and Barrientos 2013; Bikas et al. 2020).

It is known that eukaryotic cells rely on a trio of antioxidant enzymes: Cu/Zn superoxide dismutase (Sod1), catalase (Cat) and glutathione peroxidase (Gpx) to scavenge ROS produced during normal metabolism. Sod1 catalyses the conversion of the superoxide radical O_2_•- into H_2_O_2_ and oxygen; Cat subsequently breaks down H_2_O_2_ into water and molecular oxygen; and Gpx further detoxifies H_2_O_2_ and lipid peroxides using reduced glutathione as a co-factor, converting them into water and oxidised glutathione (Ighodaro and Akinloye 2018; Mattila et al. 2022; Roy et al. 2023; Jomova et al. 2024).

Additionally, we will also study novel mitonuclear combinations in hybrid strains, by analysing their mode of inheritance, levels of heterosis and the relationship between mitonuclear compatibility and mushroom productivity, in order to predict their agricultural performance. In relation to the last topic, the ability of the different strains to colonise wheat straw will be studied by analysing the expression of two genes, *lacc4* and *fet3*, which will allow the differentiation between fast- and slow-growing strains. Laccases are copper-containing oxidases, which facilitate the oxidation of phenolic substrates and can indirectly promote Fenton chemistry by generating H_2_O_2_ or by reducing Fe^3+^ to Fe^2+^. Fet3 is a ferroxidase oxidising Fe^2+^ to Fe^3+^, contributing to iron homeostasis and mitigation of oxidative stress by limiting hydroxyl radical formation through the Fenton reaction (De Silva et al. 1995; Hou et al. 2020; Amadei et al. 2025). *Fet3* and *yfh1* (a gene encoding a mitochondrial permease essential for iron–sulphur cluster assembly, haem biosynthesis and mitochondrial respiration) act together to form the high-affinity iron transport system, which is crucial for maintaining iron homeostasis and regulating the oxidative stress response (Babcock et al. 1997; Radisky et al. 1999).

We anticipate that mitonuclear incompatibilities in hybrid strains may result in exacerbated gene expression (transgressive expression) and heterosis, particularly in genes associated with respiration and the oxidative stress response. In summary, this study aims to clarify the role of mitonuclear interactions in determining hybrid fitness in basidiomycete fungi, offering valuable insights for mushroom breeding and the development of genetically stable strains.

## ﻿Materials and methods

### ﻿Fungal strains

In this study, monokaryotic (parental) and heterokaryotic (hybrid) strains of *P. ostreatus* were utilised. The hybrid strain dN001 (n), accession CECT20600 and its dedikaryotised protoclones PC9 and PC15 (Larraya et al. 1999b), were deposited in the Spanish Type Culture Collection. The strain dT009 (t) is a wild isolate collected in Alcalá de Henares (40.436614, -3.304445; Madrid, Spain). The mitochondrial genomes from the (n) and (t) strains are present in dN001 (n) and dT009 (t), respectively. Monokaryotic strains, derived from hybrid strains via meiosis, were also used (mN and mT) and their growth phenotype, fast- or slow-growing, was designated as follows: mNF / mNS (see Table 1 below).

**Table 1.** List of different strains used in this work.

Strain name	Monokaryon (m)	Heterokaryon (d)	Generation	Monokaryon number	Growth rate	Mitochondrial type
dN001 (n)		✓	F_1_	-	Fast	(n)
dT009 (t)		✓	F_1_	-	Fast	(t)
mNF418 (n)	✓		F_4_	18	Fast	(n)
mNF419 (n)	✓		F_4_	19	Fast	(n)
mNS423 (n)	✓		F_4_	23	Slow	(n)
mNS426 (n)	✓		F_4_	26	Slow	(n)
mT1 (t)	✓		F_1_	-	Fast	(t)
d(NF418 × T1, n)		✓	F_1_	-	Fast	(n)
d(NF418 × T1, t)		✓	F_1_	-	Fast	(t)
d(NF419 × T1, n)		✓	F_1_	-	Fast	(n)
d(NF419 × T1, t)		✓	F_1_	-	Fast	(t)
d(NS423 × T1, n)		✓	F_1_	-	Slow	(n)
d(NS423 × T1, t)		✓	F_1_	-	Slow	(t)
d(NS426 × T1, n)		✓	F_1_	-	Slow	(n)
d(NS426 × T1, t)		✓	F_1_	-	Slow	(t)

d: denotes a heterokaryotic strain N/T. m: denotes a monokaryotic strain meiotic-derived from the heterokaryotic strain dN/dT. F_x_ subscript refers to the generation to which the strain belongs. Monokaryon number corresponds to the identifier assigned during the isolation procedure. Mitochondrial type refers to the mitochondrial genotype harboured by each strain.

### ﻿Culture conditions

Strains were cultured and maintained on Petri dishes containing Malt Extract Solid Medium (MESM: malt extract, 20 g/l; bacteriological agar, 15 g/l). Minimal Solid Medium (MSM: 0.1 g/L Na_2_B_4_O_7_·H_2_O, 0.07 g/l ZnSO_4_·7H_2_O, 0.01 g/l CuSO_4_·5H_2_O, 0.01 g/l MnSO_4_·4H_2_O, 0.01 g/l FeSO_4_·4H_2_O, 0.01 g/l (NH_4_)_6_Mo_7_O_24_·4H_2_O; and bacteriological agar, 15 g/l), supplemented with 1% glucose, sucrose or glycerol, was used to assess the influence of carbon source on growth rates. Standard culture conditions (SC) refer to cultivation on MESM at 24 °C in darkness. Any modifications to the standard conditions are specified accordingly.

Mycelial cultures for nuclear and mitochondrial DNA extraction, as well as for gene expression analysis, were grown in Malt Extract Liquid Medium (MELM: malt extract, 20 g/l) in 250 ml Erlenmeyer flasks containing 100 ml of MELM. Mycelia were harvested after 21 days of cultivation, filtered, frozen in liquid nitrogen and stored at −80 °C until further use.

### ﻿Breeding programme aimed to develop divergent fast- and slow-growing bred lines

**Figure 1.**
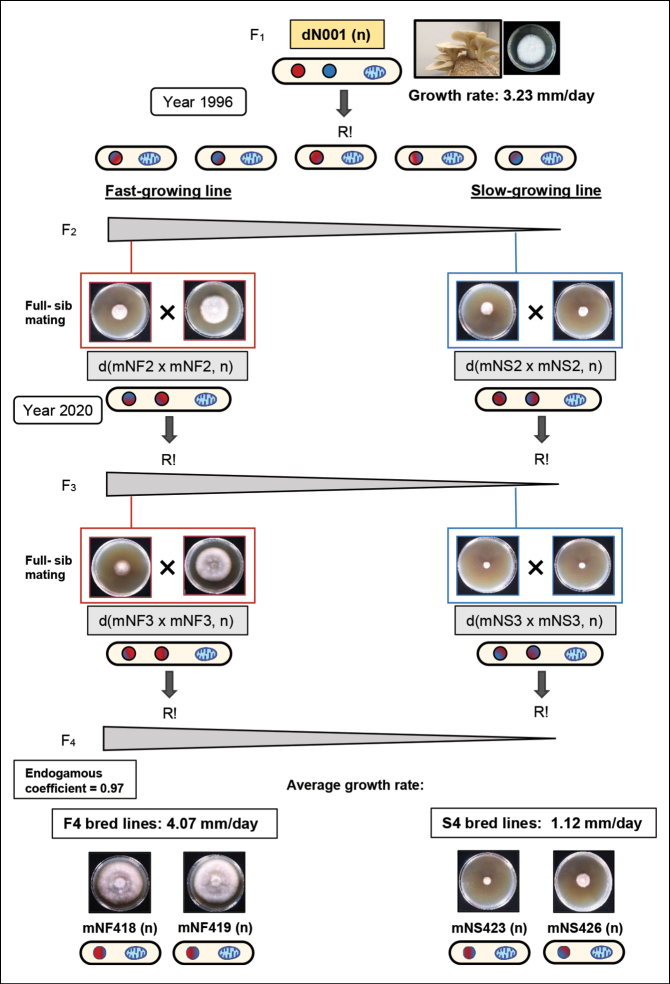
Breeding programme used to obtain divergent bred lines selected for growth rate.

Sporulation of the dN001 (n) strain gave rise to an F_2_ monokaryotic offspring ferrying an identical mitochondrial genome and exhibiting a bimodal distribution of fast- and slow-growing phenotypes. Full-sib fast-growing monokaryons were intercrossed, as were the slow-growing monokaryons, to produce advanced heterokaryotic offspring, as illustrated in Fig. 1. This breeding programme resulted in the establishment of Fast (F4) and Slow (S4) bred lines, which were used as parental strains in the present study.

### ﻿Construction of bidirectional hybrid strains

Bidirectional crosses were performed by using different monokaryon strains to obtain hybrids sharing identical nuclear genomes, but different mitochondrial type. To achieve this, a mycelial plug of 0.5 cm diameter from the F4 or S4 bred line strains was placed 30 mm from the edge of a square Petri dish. On the opposite side, a mycelial plug of the tester strain was positioned. Plates were incubated under SC, allowing the mycelia to come into contact and fully colonise the plate.

**Figure 2.**
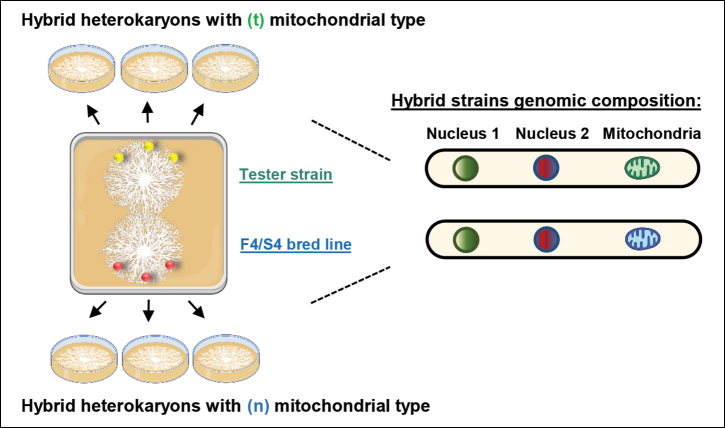
Schematic representation of bidirectional hybrid crosses and their resulting genomic composition.

A minimum of six mycelial plugs were collected from each plate as indicated in Fig. 2: three from the upper side and three from the lower side. These samples were then transferred to MESM plates and incubated until the mycelium fully colonised the plate.

Nuclear and mitochondrial molecular markers and optical microscopy were used to confirm new formed hybrids.

### ﻿Nuclear and mitochondrial DNA extraction and characterisation of molecular markers

Mycelial cultures used for the extraction of nuclear and mitochondrial DNA were grown and handled as described in Materials and Methods. Nuclear DNA was extracted using the Fungal DNA Mini Kit (Omega Bio-Tek, Norcross, GA, USA).

For mitochondrial DNA isolation, 300 mg of frozen, powdered fungal tissue were re-suspended in 500 μl of cold Mitochondrial Isolation Buffer (MIB: 0.44 M sucrose, 30 mM Tris-HCl pH 7.4, 2 mM EDTA, 1 mM phenylmethylsulphonyl fluoride [PMSF]). Samples were centrifuged at 4,000 rpm for 10 minutes at 4 °C. The supernatant was transferred to a new microcentrifuge tube and centrifuged at 16,000 rpm for 10 minutes at 4 °C. The supernatant was discarded and the pellet was immediately re-suspended in 100 μl of Solution I (25 mM Tris-HCl pH 8.0, 50 mM glucose, 10 mM EDTA pH 8.0) and 200 μl of Solution II (0.2 N NaOH, 1% SDS) and incubated on ice for 5 minutes. Subsequently, 150 μl of Solution III (5 M potassium acetate, pH 4.8) was added and the samples were placed on ice for a further 5 minutes. The mixture was then centrifuged at 13,000 rpm for 10 minutes at room temperature. The resulting supernatant was transferred to a new tube, mixed with 350 μl of phenol: chloroform: isoamyl alcohol (25:24:1) and centrifuged at 13,000 rpm for 15 minutes at room temperature. The aqueous phase was then precipitated by adding 1 ml of 100% ethanol and incubated for 10 minutes at room temperature. Pellets were re-suspended in 20 μl of TE buffer (10 mM Tris-HCl pH 8.0, 1 mM EDTA pH 8.0, containing 1.6 μg RNase) and stored at −20 °C. Three biological replicates of each strain were used for all experiments.

Nuclear and mitochondrial molecular markers were obtained following a PCR amplification reaction of a polymorphic region of chromosome VII of *P. ostreatus* and a fragment corresponding to the mitochondrial *cox1* gene used as template. PCR reactions were performed using the respective primers (Suppl. material 1: table S1).

The PCR conditions for nuclear markers were: 5 minutes at 95 °C, followed by 30 cycles of 1 minute at 95 °C, 1 minute at 62 °C and 2 minutes at 72 °C. For mitochondrial markers, the conditions were: 5 minutes at 95 °C, followed by 35 cycles of 1 minute at 95 °C, 45 seconds at 57 °C and 1 minute 30 seconds at 72 °C.

### ﻿Analysis of quantitative and molecular traits in parental and hybrid strains

#### ﻿Growth rate of parental and hybrid strains under different temperatures and carbon sources

Plugs of 0.5 cm in diameter containing actively growing mycelia from both parental and hybrid strains were used to inoculate MESM plates. Linear growth rates were recorded at 15 °C, standard conditions (SC, 24 °C) and 32 °C and measurements continued until the mycelia had fully colonised the plate. Growth rate values for each strain represent the average of three biological replicates.

To assess the influence of carbon source on fungal growth, additional measurements were carried out using MSM plates supplemented with glucose, sucrose or glycerol. Inoculated plates were incubated under SC, as described in Materials and Methods and radial growth rates were recorded in the same manner.

### ﻿Analysis of mushroom production in hybrid strains

Mycelia of hybrid strains were cultivated in glass bottles containing boiled and sterilised millet grains to produce the spawn. Growth rate values corresponded to the average of three replicates, measured as the vertical colonisation rate of the mycelia within the bottle (expressed in cm/day).

For fruiting assays, three plastic bags per strain were prepared by mixing the spawn with pasteurised wheat straw substrate at a 2% (w/w) ratio. The mixture was packed into 2 kg micro-perforated plastic bags and incubated under SC until full substrate colonisation. At this point, bags were weighed and substrate weight loss (g) was recorded. These measurements represent the average of three biological replicates.

Fruiting was induced by transferring the bags to a fruiting chamber maintained at 18 °C, 90% relative humidity and a 12 h light/dark photoperiod with continuous aeration. Data on total mushroom yield, earliness and number of flushes were recorded as was previously described (Larraya et al. 2003). Harvested fruiting bodies were stored at –80 °C for subsequent analysis.

### ﻿Qualitative determination of antioxidant compounds in fruiting bodies

For extract preparation, frozen fruiting bodies were lyophilised and ground into powder using a stomacher (Seward 400). Antioxidant compounds were extracted sequentially using both water and methanol. For the aqueous extraction, 4.5 g of powdered fruiting body was stirred with 180 ml of distilled water in an ultrasonic bath for 30 minutes. The supernatant was separated from the residue by filtration and the extraction was repeated twice more to maximise compound recovery.

For the methanolic extraction, the remaining residue was mixed with 180 ml of methanol and the same ultrasonic extraction and filtration procedure was followed. Both aqueous and methanolic extracts were concentrated using a rotary evaporator at 40 °C. Any residual water was removed by lyophilisation and the resulting powders were stored at –80 °C until further antioxidant activity assays.

The qualitative assessment of antioxidant activity in the fruiting bodies was carried out by evaluating radical scavenging activity against DPPH• (2,2-diphenyl-1-picrylhydrazyl hydrate; #D9132, Sigma-Aldrich Co., St. Louis, MO), as was described by Pinacho et al. (2015). A preliminary qualitative assessment was performed using thin-layer chromatography (TLC). Plates were developed in a chromatography chamber using two mobile phases: (i) ethyl acetate: methanol: water (65:15:5, v/v/v) and (ii) ethyl acetate: acetic acid: formic acid: water (100:11:11:26, v/v/v/v). After development, plates were air-dried at room temperature for 30 minutes until all solvents had evaporated. They were then examined under visible light and UV light (254 and 365 nm). Finally, the plates were sprayed with two reagents: (i) a DPPH solution (2 mg/ml in methanol) to detect antioxidant activity and (ii) Godin’s reagent to evaluate the relationship between antioxidant activity and chemical composition.

### ﻿ROS detection in parental and hybrid strains

Parental and hybrid strains were cultured on MESM plates until colonies reached a diameter of 30 ± 5 mm. At this stage, 10 mm diameter plugs were collected and incubated for 30 minutes at room temperature with 5 μM 2′,7′-dichlorodihydrofluorescein diacetate (H_2_DCFDA) and 10 μM dihydroethidium (DHE), both prepared in dimethyl sulphoxide (DMSO), for the detection of hydrogen peroxide (H_2_O_2_) and superoxide radicals (O_2_•-), respectively, following previous described protocols (Matzrafi et al. 2017; Romero‐Puertas et al. 2022). Three replicates per treatment were used. Exposure to light was avoided throughout incubation. Samples were subsequently washed three times with 10 mM Tris-HCl (pH 7.3).

Fluorescence was detected using a ChemiDoc Imaging System with the Alexa 488 fluorophore. All samples were exposed for equal durations. No fluorescence was observed in unstained control samples. Images were processed using Image Lab Software (2020, Bio-Rad Laboratories, Inc.) and areas of O_2_•- and H_2_O_2_ fluorescence within the plugs were quantified using ImageJ software (v1.54g, National Institutes of Health, USA).

### ﻿RNA extraction, RT-qPCR conditions and analysis of gene expression

The extraction, quantification and quality assessment of total RNA, as well as cDNA synthesis from mycelial samples, were performed as was previously described (Pérez et al. 2021). Relative gene expression level was determined by the 2^−∆∆Ct^ method using the GenEx software for processing and analysis of qPCR data (Livak and Schmittgen 2001; Castanera et al. 2013; Pérez et al. 2021). qPCR data were normalised using three reference genes (Suppl. material 1: table S1).

One-way ANOVA was employed to determine significant differences amongst parental strains, as well as between members of any hybrid strain harbouring different mitochondrial types. Differential gene expression, upregulation and downregulation, was assessed by comparing parental strains with one another and hybrids with one another.

### ﻿Sequences of genes analysed in this work

The list of primers used in this study, including those for target genes and reference genes, is provided in Suppl. material 1: table S1.

### ﻿Modes of inheritance and heterosis levels

Modes of inheritance and heterosis levels were estimated following previously described methodology (Shapira et al. 2014).

- Mid-parent value (m): The average phenotype of the two homozygous parental strains, calculated as (P _1_ + P _2_) / 2, where P _1_ and P _2_​ are the phenotypic values of the two parental strains.
- Additive genetic deviation (a): The difference between each homozygous parental phenotype and the mid-parent value, expressed as (P _1_ or P _2_) − m.
- Dominant genetic deviation (d): The deviation of the heterozygous hybrid phenotype (F _1_) from the mid-parent value, calculated as F _1_ − m.

The degree of dominance was determined by the ratio *d/a*, which was used to classify the mode of inheritance:

- *d/a = 0*: co-dominance;
- *0 < d/a < 1* or −*1 < d/a < 0*: partial dominance of the higher- or lower-value parent, respectively;
- *d/a = 1* or −*1*: complete dominance of the higher- or lower-value parent, respectively;
- *d/a > 1* or *d/a < −1*: overdominance or underdominance of the hybrid, respectively.

Mid-Parent Heterosis (MPH) compares the hybrid performance to the average performance of both parental strains:

MPH = [(F_1_ - m) / m] * 100

In hybrid strains, we examined the mode of inheritance of gene expression in relation to the expression levels of the superior parent and the corresponding heterosis values.

### ﻿Statistical analysis

Statistical analyses of three replicates data for growth rate, total mushroom yield and its components, ROS levels and gene expression were performed using IBM SPSS Statistics, version 28.0.1.1 (IBM Corporation 2021). One-way analysis of variance (ANOVA) followed by Scheffe’s post hoc multiple comparison test was used to evaluate differences amongst parental and hybrid strains. Statistical significance was defined as *P*-value < 0.05.

### ﻿Declaration of AI-assisted technologies in the writing process

During the preparation of this work, the authors used ChatGPT to assist with revising the English syntax and orthography. After utilising this tool, the authors thoroughly reviewed and edited the content as necessary and take full responsibility for the published article’s content.

## ﻿Results

### ﻿Molecular markers for the identification of nuclear and mitochondrial genomes

In this paper, we used nuclear and mitochondrial molecular markers to identify parental and hybrid genomes as well as their mitochondrial types. Both nuclear genomes in *P. ostreatus* were analysed using a primer pair (see Suppl. material 1: table S1) targeting a region on chromosome VII. This region is polymorphic between the PC9 and PC15 protoclones due to the presence of a MULE transposon insertion in the PC15 genome (Castanera et al. 2016). As result, PCR amplification produced a 0.4 kb fragment in the fast-growing bred lines and a 3.6 kb fragment in the slow-growing ones. For the tester strain mT1 (t), a 0.3 kb band was obtained (see Suppl. material 1: figs S1, S2).

In hybrids, uniparental mitochondrial inheritance was demonstrated by the presence of a single mitochondrial genome type, either (n) or (t). Amplification was carried out using a primer pair targeting the mitochondrial *cox1* gene (see Suppl. material 1: table S1), yielding two distinct fragment sizes: a 1.9 kb fragment for strains d(NF418 × T1, n), d(NF419 × T1, n), d(NS423 × T1, n) and d(NS426 × T1, n); and a 0.2 kb fragment for the corresponding (t) variants of these strains (see Suppl. material 1: fig. S3).

### ﻿A divergent selection programme aimed at maximising phenotypic variation in growth rate

With the purpose of determining the influence of an identical mitochondrial type on fast- and slow- growing monokaryons (F_2_, F_n_) offspring derived from the dN001 (n) hybrid strain, we performed (as was described in Materials and Methods) mating designs which facilitated the accumulation of alleles associated with either fast or slow growth rates in the F4 and S4 bred lines (which exhibited an inbreeding coefficient of 0.97) with average growth rates of 4.07 mm/day and 1.12 mm/day, respectively (see Fig. 1).

The Selection Response Coefficient (R = x̄ F_4_ − x̄ F_2_) yielded a positive value of 2.03 mm/day for the fast-growing lines and a slightly negative value of −0.02 mm/day for the slow-growing ones (see Suppl. material 1: table S2). These values represent a 50% increase in growth rate for the fast-growing lines and a 1.8% decrease for the slow-growing ones.

Results obtained showed: i) a clear genotypic difference between the two bred line types and ii) suggest that the reduced growth observed in certain lines may, amongst other contributing factors, result from the disruption of co-evolved mitonuclear interactions.

### ﻿Construction of hybrid strains

The breeding programme yielded thirty fast-growing (F4) and thirty slow-growing (S4) bred lines which were evaluated using five different tester strains to assess their General Combining Ability (GCA) and to generate F_1_ hybrids exhibiting enhanced hybrid vigour. Amongst the testers used, only strain mT1 (t) (derived from the strain dT009 (t)) was compatible with all selected bred lines, successfully forming hybrids containing either the (n) or (t) mitochondrial type. Consequently, four fast-growing and four slow-growing hybrids were obtained (see Table 1). Half of these hybrids carried the (n) mitochondrial genome, derived from the hybrid strain dN001 (n), while the other half inherited the (t) type from the tester strain mT1(t) (see Fig. 2).

### ﻿Analysis of growth rate under standard conditions (SC, 24 °C) and at 15 °C and 32 °C in parental and hybrid strains

**Figure 3.**
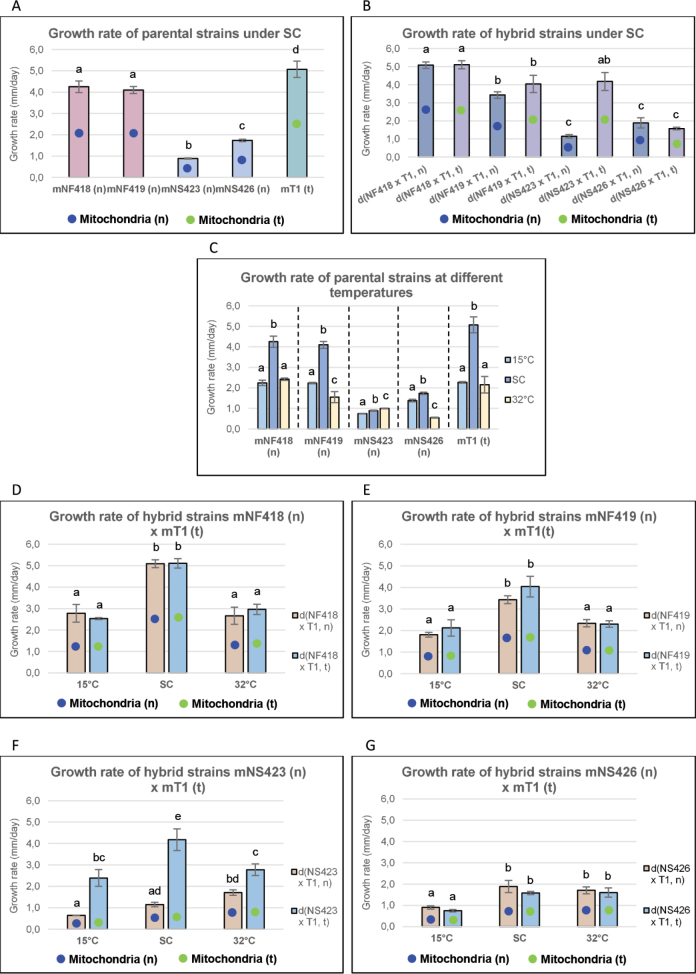
Growth rate under SC conditions and at different temperatures. Growth rate of fast- and slow-growing parental and hybrid strains under SC conditions in (A, B), respectively and at different temperatures in (C, D, E, F, G). Bar length represents the average of three replicates. Blue and green colours indicate the (n) and (t) mitochondrial types, respectively.

The performance of parental and hybrid strains under SC (24 °C) is illustrated in Fig. 3A, B. Significant differences were observed between the fast-growing and slow-growing bred lines, as well as between their respective hybrids. Two points merit particular attention: the notably higher growth rate of the tester strain mT1(t) compared to other parental strains and the significant growth rate difference between the hybrid strains d(NS423 × T1, t) and d(NS423 × T1, n).

When parental strains were cultured at 15 °C and 32 °C (Fig. 3C, Suppl. material 1: fig. S4A, table S3.1), it was evident that strains mNF418 (n) and mT1(t) exhibited similar reductions in growth rate (approximately 40–60%) compared to their optimum performance at SC. A similar pattern of reduction was also observed in the fast-growing bred line mNF419 (n) and the slow-growing line mNS426 (n); however, their growth rates were less affected at 15 °C than at 32 °C relative to SC.

The strain mNS423 (n) showed only around a 15% decrease at 15 °C and a 10% increase at 32 °C compared to SC, indicating a stable growth performance across the temperature range tested (Fig. 3C, Suppl. material 1: fig. S4A, table S3.1).

Results derived from the parental strains suggest that the observed differences arise from genotype-by-environment interactions, specifically (G_1_, n) × E_1_…E_n_ and (G_2_, t) × E_1_…E_n_ genome interactions and point to the disruption of co-evolved mitonuclear interactions as the cause of hybrid breakdown in the slow-growing bred lines.

Analysis of the fast-growing hybrid strains d(NF418 × T1, n/t) and d(NF419 × T1, n/t) revealed reduced growth rates at both 15 °C and 32 °C compared with the SC, with heterosis values ranging from 16% to 30% in d(NF418 × T1, n/t) at 32 °C (see Suppl. material 1: table S4. MPH1) and around 25% in d(NF419 × T1, n/t), suggesting influence of temperature, but not of mitochondrial type (Fig. 3D, E, Suppl. material 1: fig. S4B, table S3.2).

The slow-growing hybrids d(NS423 × T1, n/t) exhibited variation across temperature conditions (Fig. 3F, Suppl. material 1: fig. S4B, table S3.2). The d(NS423 × T1, t) strain displayed higher growth rates than d(NS423 × T1, n) at all temperatures, accompanied by high heterosis values ranging from 40% to 76% (Suppl. material 1: table S4. MPH1), suggesting that mitonuclear genomic interactions, influenced by temperature, contribute to these differences. No mitochondrial effect was detected in the growth rates of strains d(NS426 × T1, n/t), although both variants displayed reduced growth at 15 °C (Fig. 3G, Suppl. material 1: fig. S4B, table S3.2).

In summary, it should be noted that the growth performance of the parental strains and their hybrids depends on mitonuclear genome interactions influenced by temperature. Accordingly, growth rate in the parental strains seems to be dependent on (G_1_, n) × E_1_…E_n_ and (G_2_, t) × E_1_…E_n_ interactions, while, in the hybrids, it depends on (G_1_, G_2_, n/t) × E_1_…E_n_ interactions.

### ﻿Analysis of growth rate on different carbon sources in parental and hybrid strains

**Figure 4.**
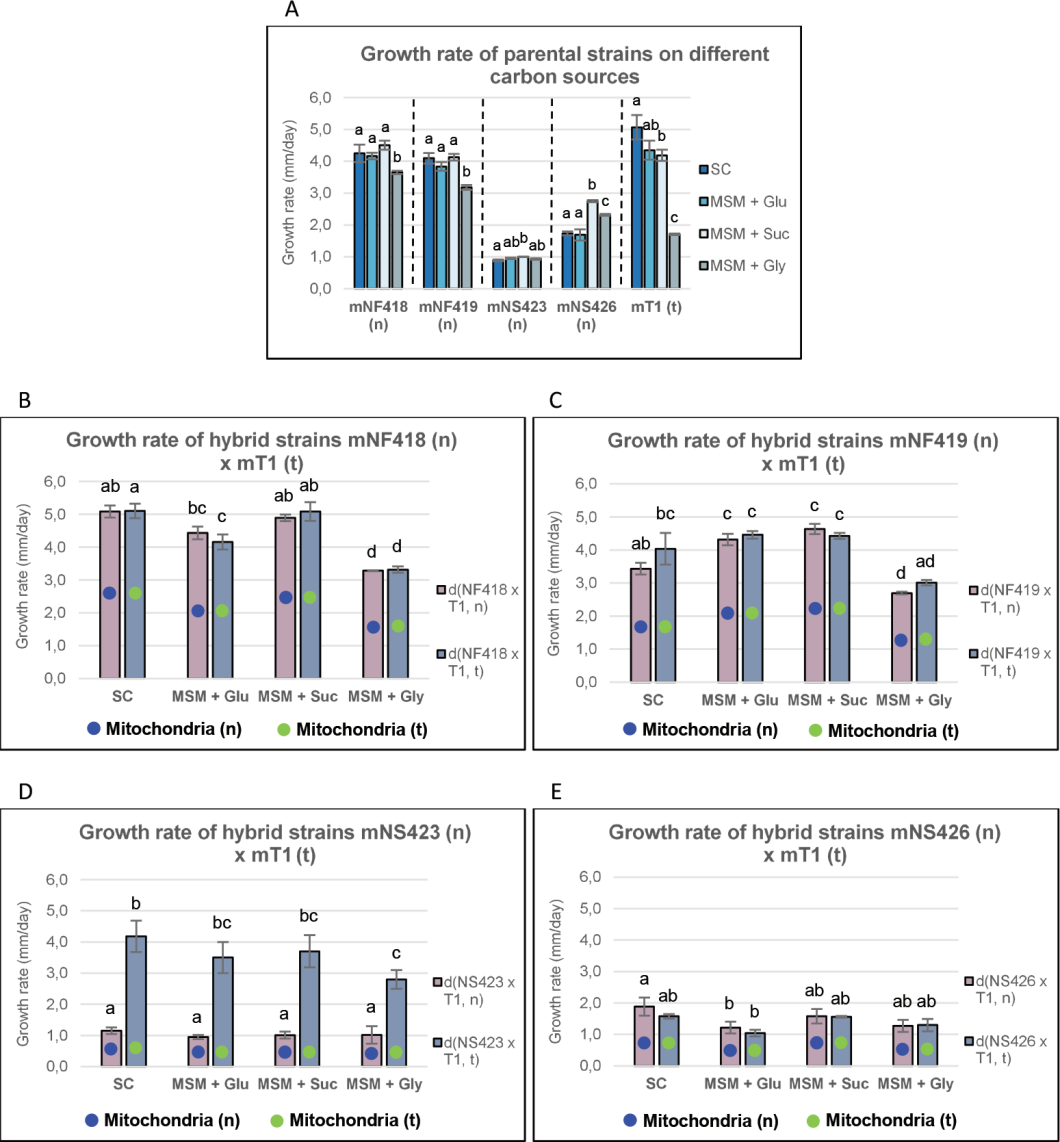
Growth rate on different carbon sources. Growth rate of parental (A) and hybrids strains (B, C, D, E) grown on different carbon sources. MSM + Glu: MSM culture medium supplemented with glucose; MSM + Suc: MSM culture medium supplemented with sucrose; MSM + Gly: MSM culture medium supplemented with glycerol. Bar length represents the average of three replicates. Blue and green colours indicate the (n) and (t) mitochondrial types, respectively.

We observed that growth rate of the F4 bred lines and the tester strain mT1(t) was largely unaffected by the carbon sources supplemented in the MSM culture medium. The only exception was MSM supplemented with glycerol, which caused a notable reduction in growth (Fig. 4A, Suppl. material 1: fig. S5A, table S3.1). A similar pattern was observed in the tester strain mT1(t), whose growth rate on this medium decreased by approximately 60%. This strain also showed a slight reduction in growth when grown on MSM supplemented with sucrose compared to growth at SC.

MSM supplemented with sucrose was the most favourable fermentable carbon source for the growth of S4 bred lines. However, the strain mNS423 (n) maintained consistent growth rates across all tested carbon sources (Fig. 4A, Suppl. material 1: fig. S5A, table S3.1).

Fast-growing hybrids d(NF418 × T1, n/t) and d(NF419 × T1, n/t), cultured on different carbon sources (Fig. 4B, C, Suppl. material 1: fig. S5B, table S3.2), showed no influence of mitochondrial type on growth rate. However, when grown on MSM supplemented with glycerol, a reduction in growth rate and heterosis values between 10–24% were observed with influence of the mitochondrial type in the hybrids d(NF419 × T1, n/t) (see Suppl. material 1: table S5. MPH2), indicating a (G_1_, G_2_, n/t) × E_1_ interaction.

The hybrid strain d(NS423 × T1, t) (Fig. 4D, Suppl. material 1: fig. S5B, table S3.2) exhibited a growth pattern on different carbon sources similar to that observed at varying temperatures, with heterosis values ranging from 32–100% depending on the carbon source and mitochondrial type (see Suppl. material 1: table S5. MPH2). This result highlights the significant role of the (t) mitochondrial type in enhancing growth rate across different temperatures and carbon sources, revealing the presence of (G_1_, G_2_, t) × E_1_…E_n_ and (G_1_, G_2_, t) × J_1_…J_n_ interactions.

### ﻿Expression analysis of the ETC and detoxification-related genes in parental and hybrid strains

We analysed gene expression by RT-qPCR in both parental and hybrid strains, focusing on genes involved in the ETC and detoxification pathways. For the ETC, we examined genes encoding proteins associated with Complex I: NADH dehydrogenase subunit 1 (*nd1*, mitochondrial-encoded gene); Complex III: the mitochondrial AAA protein (ATPase Associated with Diverse Cellular Activities; (*bcs1*), E.C. 3.6.1, ID 1105500) and the Rieske iron–sulphur protein (*rip1*) (E.C. 7.1.1.8, ID 1088400); and Complex IV: cytochrome c oxidase subunits 4 (*cox4*) and 5b (*cox5b*) (E.C. 7.1.1.9, IDs 1087668 and 1094413, respectively) (Burke et al. 1997; Wirth et al. 2016; Ndi et al. 2018; Douiev et al. 2021; Hou et al. 2023). Additionally, genes involved in detoxification were assessed, including Cu/Zn superoxide dismutase (*sod1*) (E.C. 1.15.1.1, ID 1113505), catalase (*cat*) (E.C. 1.11.1.6, ID 1090819) and glutathione peroxidase (*gpx*) (E.C. 1.11.1.9, ID 1090305) (Ighodaro and Akinloye 2018; Roy et al. 2023; Jomova et al. 2024).

### ﻿Expression analysis of genes belonging to different complexes of the ETC

#### ﻿Complex I – *nd1* expression

**Figure 5.**
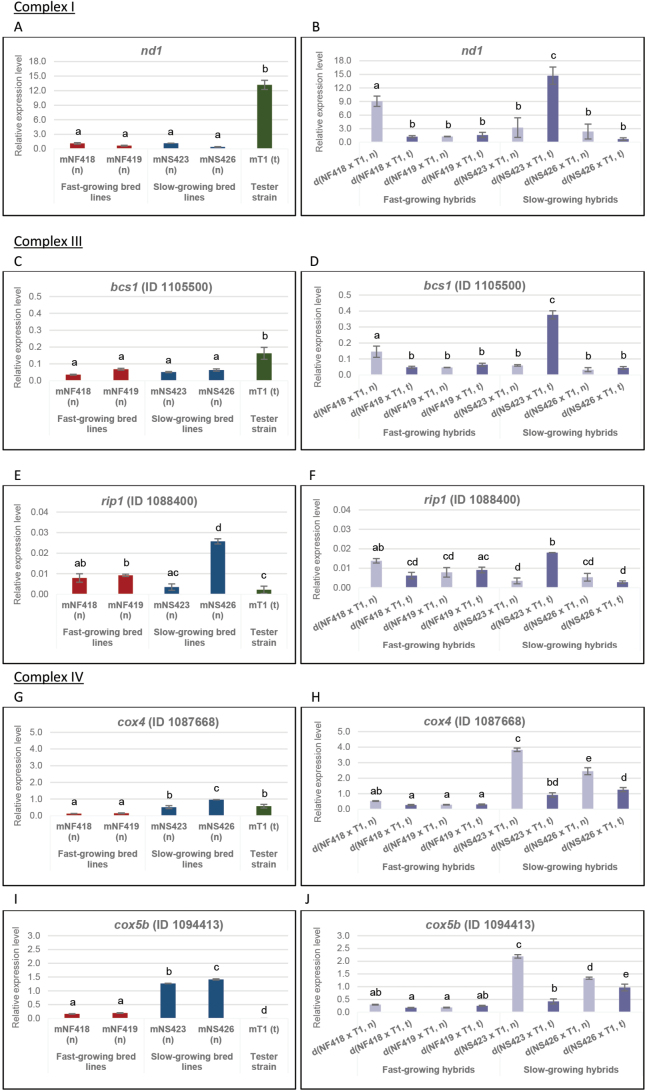
Relative expression levels of ETC genes. Relative expression levels of genes associated with ETC complexes in parental (A, C, E, G, I) and hybrid strains (B, D, F, H, J). Bar length represents the average of three replicates.

No significant differences in *nd1* expression were observed between the fast- and slow-growing bred lines (Fig. 5A); however, mitochondrial type had a marked effect in some of the hybrid strains. For example, the strains d(NF418 × T1, n) and d(NS423 × T1, t) showed upregulation of the *nd1* gene according to their mitochondrial type (see Fig. 5B), indicating mitonuclear interactions. The mode of inheritance of the *nd1* gene in both hybrids was partial dominance and overdominance. The observed overdominance reflects the epistatic effect of the mT1 (t) parental line on the d(NS423 × T1, t) hybrid, with a heterosis value of 100% (see Suppl. material 1: tables S1. MI1, S6. MPH3).

#### ﻿Complex III – *bcs1* and *rip1* expression

Upregulation of both genes in the hybrids d(NF418 × T1, n) and d(NS423 × T1, t) suggests an improved oxygen-dependent environment compared to d(NF418 × T1, t) and d(NS423 × T1, n) (Fig. 5D, F). The influence of the mitochondrial type on the expression profiles of these genes in the strains d(NF418 × T1, n) and d(NS423 × T1, t), their modes of inheritance (partial dominance and overdominance for *bcs1* and overdominance for *rip1*) and heterosis values (see Fig. 5D, F, Suppl. material 1: tables S2. MI2, S3. MI3, S7. MPH4, S8. MPH5) followed a pattern similar to that observed for the *nd1* gene.

#### ﻿Complex IV – *cox4* and *cox5b* expression

Expression levels of *cox4* and *cox5b* differentiated fast- and slow- growing bred lines and their hybrids (see Fig. 5G–J). The upregulation of both genes in the slow-growing bred lines (Fig. 5G, I) suggests the occurrence of oxidative stress and hypoxia which could be the result of the disruption of co-evolved mitonuclear interactions, thereby impairing ETC function. The slow-growing hybrid strains d(NS423 × T1, n) and d(NS426 × T1, n) (see Fig. 5H, J) showed upregulation of both genes. In the hybrid d(NS423 × T1, n), the mode of inheritance of the *cox4* gene was overdominance. Additionally, the *cox5b* gene showed this type of mode of inheritance. In the hybrid d(NS426 × T1, n), the *cox4* gene exhibited overdominance, while partial dominance was observed for the *cox5b* (Suppl. material 1: tables S4. MI4, S5. MI5). High heterosis values were influenced by the mitochondrial type (see Suppl. material 1: tables S9. MPH6, S10. MPH7). However, downregulation of both genes in the strain d(NS423 × T1, t) to levels comparable with fast-growing hybrids (see Fig. 5H, J) indicates that the presence of the (t) mitochondrial type contributes to reducing oxidative stress and thus hypoxia. The modes of inheritance were overdominance for *cox4* and partial dominance for *cox5b* (Suppl. material 1: tables S4. MI4, S5. MI5).

### ﻿Analysis of the expression profiles of detoxification-related genes

**Figure 6.**
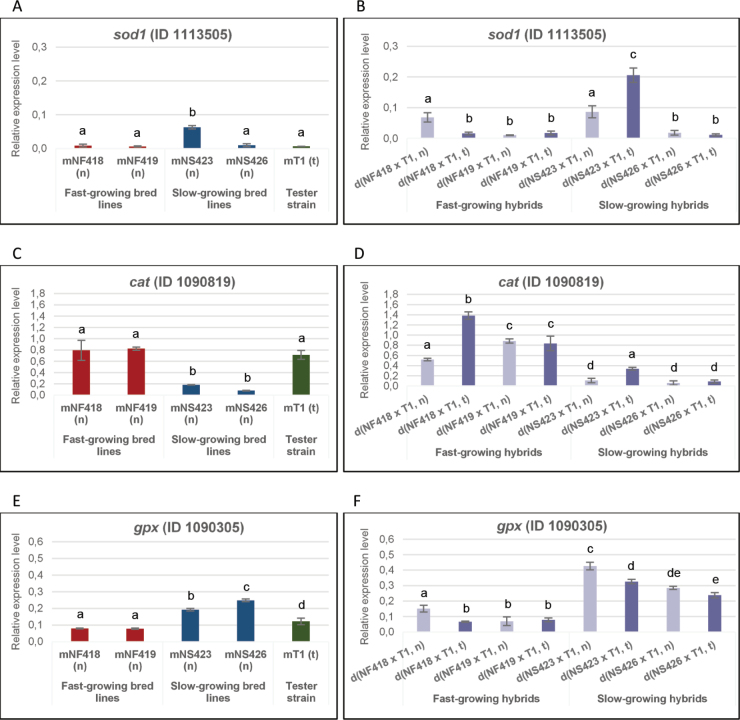
Relative expression levels of genes encoding detoxifying enzymes. Relative expression levels of detoxification genes in parental (A, C, E) and hybrid strains (B, D, F). Bar length represents the average of three replicates.

Analysis of *sod1* gene expression amongst parental lines revealed no significant differences, except for the mNS423 (n) strain, whose upregulation suggests high levels of oxidative stress (see Fig. 6A). In the hybrid strains, notable variation of *sod1* gene expression was observed in d(NF418 × T1, n/t) and d(NS423 × T1, n/t) (Fig. 6B). These differences appear to be influenced by the mitochondrial type and could reflect transcriptional dysregulation resulting from mitonuclear genome conflict. At least three out of the six hybrid strains exhibited elevated expression levels, indicative of a high oxidative stress (Fig. 6B). Overdominance was the mode of inheritance for the *sod1* gene in all hybrids (Suppl. material 1: table S6. MI6), which also showed high heterosis values influenced by the mitochondrial type (Suppl. material 1: table S11. MPH8).

A combined analysis of *cat* and *gpx* gene expression profiles enabled differentiation between fast- and slow-growing bred lines and their hybrids (Fig. 6C–F). In fast-growing strains, *cat* was upregulated, while *gpx* was downregulated, with the reverse pattern seen in slow-growing strains.

Significant differences in expression of both genes were observed in hybrids d(NF418 × T1, n/t) and d(NS423 × T1, n/t), dependent on the mitochondrial type, mirroring the pattern seen for *sod1*. The mode of inheritance for *cat* and *gpx*, underdominance versus overdominance (or vice versa) and the elevated heterosis values in d(NF418 × T1, n/t) hybrids corresponded to the mitochondrial type (see Suppl. material 1: tables S7. MI7, S8. MI8, S12. MPH9, S13. MPH10). A similar pattern was observed in d(NS423 × T1, n/t) hybrids, with underdominance and partial dominance as modes of inheritance for *cat* and overdominance for *gpx*, with heterosis exceeding 100% in both hybrids (see Suppl. material 1: tables S7. MI7, S8. MI8, S13. MPH10).

In summary, it may be concluded that the transgressive inheritance observed in hybrids’ detoxification-related genes is likely associated with one of the parental strains and influenced by the mitochondrial type.

### ﻿Determination of oxidative stress by analysing ROS using probes detecting H_2_O_2_/peroxides in parental and hybrid strains

**Figure 7.**
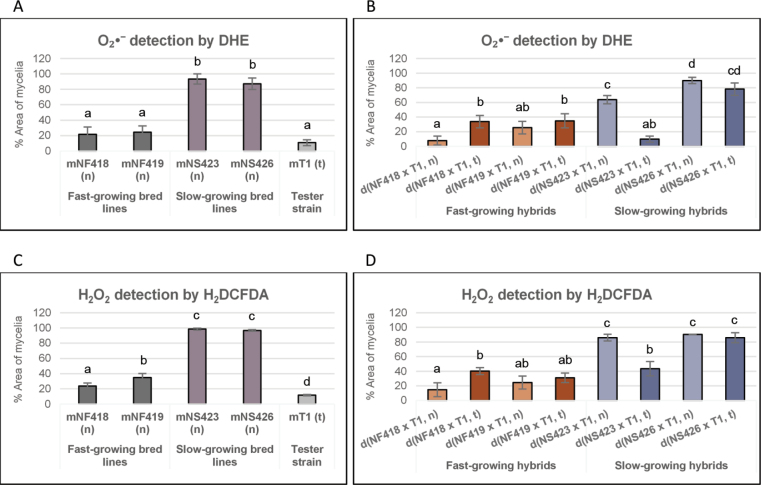
ROS detection. ROS levels in parental and hybrid strains are expressed as the percentage of mycelial area stained by DHE (A, B) and H_2_DCFDA (C, D). Bar length represents the average of three replicates.

We used two fluorogenic probes (DHE for superoxide O_2_•- detection and H_2_DCFDA for H_2_O_2_ detection) to assess ROS levels in both parental strains and their hybrids (see Materials and Methods). The analysis revealed a high accumulation of ROS in slow-growing bred lines and their hybrids compared to the fast-growing strains (Fig. 7A–D).

Notably, the hybrid d(NS423 × T1, t) exhibited a marked reduction in ROS levels (both O_2_•- and H_2_O_2_) comparable to those observed in fast-growing hybrids (Fig. 7B, D). This decrease in ROS was consistent with the expression profiles of the antioxidant-encoding genes described above (Fig. 6B, D, F). The data suggest that the (t) mitochondrial type contributes to reducing oxidative stress in the d(NS423 × T1, t) hybrid, as evidenced by the downregulation of *cox4* and *cox5b* (Fig. 5H, J).

### ﻿Analysis of the influence of the mitochondrial genome on quantitative traits related to mushroom production in hybrid strains

Building on the remarkable performance observed in the d(NS423 × T1, n/t) hybrids, we further investigated their behaviour under industrial cultivation conditions. The aim was to assess the influence of the mitochondrial genome on key quantitative traits related to mushroom production such as: spawn colonisation, substrate weight loss, total mushroom yield and its components: earliness and number of flushes.

**Figure 8.**
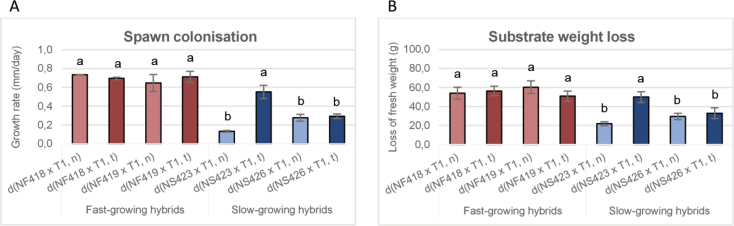
Spawn colonisation (cm/day) (A) and substrate weight loss (g) (B) in fast- and slow-growing hybrid strains. Bar length represents the average of three replicates.

Spawn colonisation was assessed by measuring mycelial growth rates of the hybrid strains. No significant effect of mitochondrial type on growth rates was observed in either the fast-growing hybrids or in the slow-growing d(NS426 × T1, n/t) hybrid. However, the presence of the (t) mitochondrial type in d(NS423 × T1, t) hybrid resulted in a hybrid with a growth rate comparable to that of the fast-growing hybrids (see Fig. 8A).

Subsequent cultivation was performed in plastic bags containing straw-based substrate. Substrate weight loss, used as a proxy for substrate degradation efficiency, was measured. The results mirrored those of spawn colonisation: d(NS423 × T1, t) exhibited substrate weight loss similar to the fast-growing hybrids, indicating a strong correlation between these traits (Fig. 8B).

Results obtained underscore the significant influence of mitochondrial type on both growth rate and substrate degradation capacity in the d(NS423 × T1, n/t) hybrids, where the (t) mitochondrial inheritance appears to be involved in enhancing growth performance under industrial cultivation conditions.

We then analysed the total mushroom yield and its components, such as earliness and the number of flushes. The components contributing to total mushroom yield were measured as outlined in Materials and Methods. As with growth rate, the performance of hybrid strains depended on genotype. The mitochondrial type had a significant effect in the hybrid d(NS423 × T1, t), whose total mushroom yield and component values were similar to those of fast-growing hybrids (see Table 2). These traits were not influenced by the mitochondrial type in fast-growing hybrids.

**Table 2.** Mushroom production and its components. Components of mushroom production and total yield in fast- and slow-growing hybrid strains. Values are presented as the mean of three replicates ± Standard Deviation (SD).

Hybrid strains	Earliness (days)	Number of flushes	Total mushroom yield (g)
Fast-growing hybrids			
d(NF418 × T1, n)	13 ± 2^a^	3 ± 1^a^	299.2 ± 77.2^a^
d(NF418 × T1, t)	13 ± 1^a^	4 ± 0^a^	256.0 ± 41.5^ab^
d(NF419 × T1, n)	14 ± 3^a^	4 ± 1^a^	199.7 ± 15.1^bc^
d(NF419 × T1, t)	14 ± 0^a^	4 ± 1^a^	211.8 ± 36.7^bc^
Slow-growing hybrids			
d(NS423 × T1, n)	38 ± 3^b^	1 ± 0^b^	29.2 ± 5.0^d^
d(NS423 × T1, t)	26 ± 1^c^	3 ± 0^a^	148.2 ± 11.6^c^
d(NS426 × T1, n)	35 ± 1^b^	1 ± 1^b^	39.4 ± 12.4^d^
d(NS426 × T1, t)	36 ± 2^b^	1 ± 0^b^	35.4 ± 13.0^d^

Different letters indicate significant differences.

Overall, these findings indicate that, in the d(NS423 × T1, t) hybrid, quantitative traits such as spawn colonisation, substrate degradation, earliness, number of flushes and total mushroom yield are influenced by mitochondrial type. This highlights the critical role of mitonuclear genome compatibility in maximising mushroom production.

Results obtained in this study showed that the hybrid strain d(NS423 × T1, t) exhibited higher straw-based substrate weight loss comparable to that of the fast-growing hybrids (Fig. 8B). As substrate degradation involves numerous enzymes, including laccases, we analysed the expression profile of *lacc4* (E.C.1.10.3.2, ID 1077328) and *fet3* (formerly *lacc5*, E.C.1.10.3.2, ID 1094975) in parental strains and their hybrids because both genes allowed differentiation between fast- and slow-growing strains.

**Figure 9.**
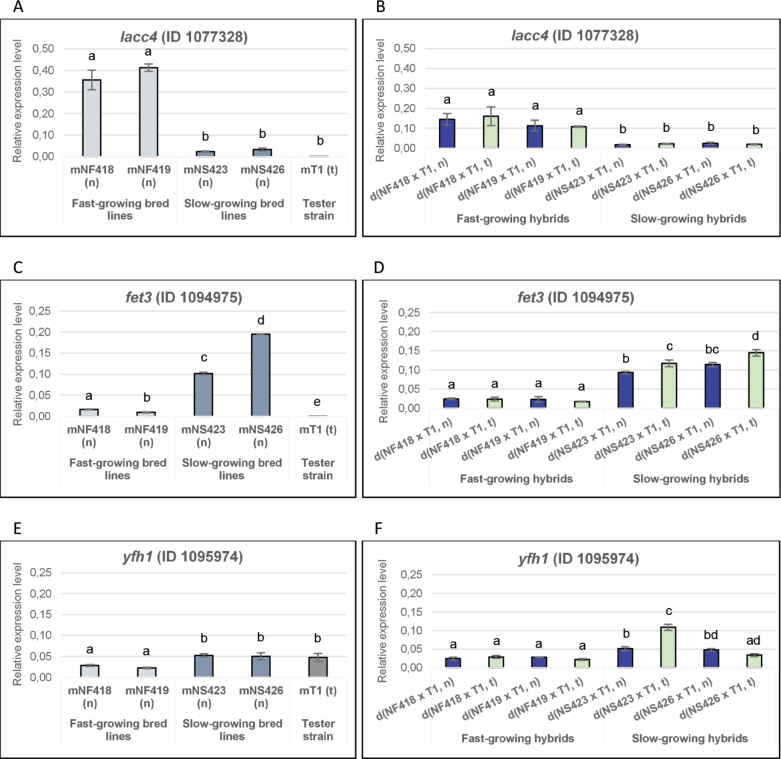
Relative expression levels of genes associated with substrate colonisation, oxidative stress and iron homeostasis. Relative expression levels of genes in parental (A, C, E) and hybrid strains (B, D, F). Bar length represents the average of three replicates.

We observed that *lacc4* gene expression was higher in the fast-growing bred lines and their hybrids than in the slow-growing ones, whereas *fet3* expression was highest in the slow-growing parental and hybrid strains. The tester strain mT1(t) exhibited *lacc4* expression levels similar to those of the slow-growing parental strains and there was no detectable *fet3* expression (see Fig. 9A–D). Interestingly, in the slow-growing hybrids, the *fet3* gene expression pattern resembled that of *cox4*, *cox5b* and *gpx* genes involved in the defence against oxidative stress and hypoxia. An exception was observed in the d(NS423 × T1, t) hybrid, where the (t) mitochondrial genotype appeared to mitigate oxidative stress and enhance the antioxidant response via *fet3* upregulation. This increased expression may have contributed to its higher growth rate in comparison with that of d(NS423 × T1, n).

We also analysed the expression of the *yfh1* gene (E.C.1.16.3.1, ID 1095974), which was significantly higher in the slow-growing bred lines and in the tester strain than in the fast-growing bred lines (see Fig. 9E). In the d(NS423 × T1, t) hybrid, the *yfh1* expression pattern suggested a partial recovery of iron availability (homeostasis) and respiratory efficiency through hybridisation (Fig. 9F).

Given that the fruiting bodies of *P. ostreatus* are known to contain high levels of antioxidant compounds, we evaluated their presence in the fruiting bodies of hybrid strains using two different extraction methods (water and methanol), two mobile phases (65:15:5 and 100:11:11:26) and different detection reagents. DPPH was used to identify antioxidant compounds, while Godin’s reagent was employed to detect secondary metabolites such as terpenes, polyphenols, flavonoids and secoiridoids (see Materials and methods).

Amongst the six possible combinations tested, one (comprising aqueous extraction, the 100:11:11:26 mobile phase and DPPH as the staining reagent) successfully detected antioxidant compounds with variable polarity in both fast- and slow-growing hybrids (Suppl. material 1: fig. S6A, C). A distinguishing feature of this combination was a band at Rf = 0.80, observed exclusively in fast-growing hybrids with the (n) mitochondrial type.

The second combination (aqueous extract, the 100:11:11:26 mobile phase and Godin’s staining reagent) revealed less polar, terpenoid-like profiles in both fast- and slow-growing hybrids, with the exception of the d(NF418 × T1, n) strain, which exhibited an additional band at Rf = 0.87 (Suppl. material 1: fig. S6B, D).

Taken together, results obtained showed that the combination of aqueous extraction, the 100:11:11:26 mobile phase and DPPH staining proved to be effective in distinguishing between fast- and slow-growing hybrids. Additionally, it also permitted the identification of fast-growing hybrids with the (n) mitochondrial type.

## ﻿Discussion

Extended research conducted in Burton’s lab using *Tigriopus californicus* (Burton 1990a, 1990b; Burton et al. 2006; Ellison and Burton 2008b; Healy and Burton 2020) demonstrated that disrupting co-evolved mitonuclear interactions resulted in mismatches between nuclear and mitochondrial genes responsible for essential cellular functions such as transcription and translation (Ellison and Burton 2006, 2008a; Burton et al. 2013). These disruptions primarily led to: i) altered expression of ETC genes involved in cellular ATP production; ii) upregulation of stress-response pathways due to exacerbated oxidative stress and iii) dysregulation of gene expression, which appeared in hybrids as transgressive expression (Ellison and Burton 2006, 2008b; Barreto and Burton 2013; Barreto et al. 2015; Healy and Burton 2020; Han and Barreto 2021).

In this study, we examined the influence of mitonuclear interactions in hybrids of the edible, lignin-degrading and model fungus *P. ostreatus* maintained in subculture. Previous results in our group showed that the commercial hybrid strain dN001 (n), as well as its bimodal (fast- and slow-growing) monokaryotic offspring, experienced a 50% reduction in growth rate over the past 20 years (Pérez et al. 2021). This reduction was associated with oxidative stress detected in the slow-growing offspring, which exhibited symptoms of strain degeneration and upregulation of genes related to the ETC, stress response, hypoxia, detoxifying enzymes, apoptosis, cell wall remodelling and lipid metabolism, amongst others (Pérez et al. 2021). These findings, in our opinion, parallel the hybrid breakdown observed in advanced generations of diploid organisms, in which co-evolved mitonuclear interactions are disrupted (Burton et al. 2006). For this reason, we developed a divergent breeding programme using the hybrid strain dN001 (n), based on matings between full-sib monokaryons sharing an identical mitochondrial type, but differing in growth rate. The increased growth rate observed in the F4 bred lines suggests that co-evolved mitonuclear interactions were maintained in these genotypes. Furthermore, it also indicates that the high-yield mushroom production observed in the dN001 (n) hybrid strain may be associated with the PC9 protoclone genome and its (n) mitochondrial type. In fact, this assumption is supported by previously published data from our laboratory, which identified in PC9 and fast-growing monokaryons, genes that were upregulated and positively correlated with growth rate, such as those encoding integral components of the mitochondrial membrane (ETC), as well as ribosomal proteins and components of the transcription and translation machinery (Pérez et al. 2021).

Growth rate in advanced generations and in the bred lines F4 and S4, grown under SC, was genotype-dependent. The occurrence of fast- and slow-growing bred lines (mNF419 (n) and mNS426 (n)) with similar growth rate reductions at 15 °C and 32 °C demonstrated that distinct genomic regions regulate growth rate and temperature sensitivity. The slow-growing bred line mNS423 (n) appeared to be well adapted to temperature, exhibiting stable growth rate performance across all tested temperatures. Taken together, these results indicate mitonuclear genome plus environment interactions, (G_1_, n) x E_1_…E_n_ in parental strains and suggest that differences observed in fast- and slow-growing bred lines could result from a lack of co-evolved mitonuclear interactions in the slow-growing bred lines as was observed previously in *Tigriopus californicus* and in the catfish belonging to genus *Ictalurus* (Ellison and Burton 2008b; Wang et al. 2022).

In the slow-growing hybrid d(NS423 × T1, t), we observed consistently high growth rate heterosis across all temperatures. This finding could be interpreted as a result of epistatic interactions between both nuclear genomes and the mitochondrial type. Alternatively, it may be a consequence of disruptions in regulatory cellular mechanisms arising from genetic incompatibilities between the hybrid parental genomes and their interactions with the mitochondrial genome. These disruptions likely affect mechanisms that typically restrict cell growth to protect against damage or prepare for stress, as reported by Herbst et al. in budding yeast hybrids (Herbst et al. 2017).

The growth rates of F4 and S4 bred lines revealed that they grew well on all the tested fermentable carbon sources. Notably, the growth rate of the strain mNS423 (n) differed from that of the other parental lines, reflecting its unique genome composition that allows a flexible carbohydrate metabolism, as demonstrated by its consistent growth across the various carbon sources tested.

The performance of the slow-growing hybrid d(NS423 × T1, t) was distinctive and consistent with observations made when this strain was grown at different temperatures and on different carbon sources, emphasising the importance of the (t) mitochondrial type in increasing its growth rate across different temperatures and carbon sources. This finding revealed (G_1_, G_2_, t) x E_1_…E_n_ and (G_1_, G_2_, t) x J_1_…J_n_ interactions underlying this special phenotype, which could result from physiological dysfunction due to incompatibilities between nuclear and mitochondrial genomes (Wang et al. 2024).

Taken together, these results allow us to conclude that, in the model organism *P. ostreatus*, strain growth rate depends on both its nuclear and mitochondrial genomes, as well as on their interaction with environmental variables such as temperature and carbon sources.

Aerobic energy production relies on the oxidative phosphorylation (OXPHOS) pathway, which depends heavily on the coordination amongst 13 proteins encoded by mitochondrial DNA and the majority of proteins encoded by nuclear DNA. Mutations that disrupt any OXPHOS components can inhibit ATP synthesis and increase oxidative stress (Barreto and Burton 2013).

The upregulation of genes within various ETC complexes can be influenced by multiple factors, such as the cell’s energy requirements, environmental changes and the need to maintain mitochondrial function during stress. Variations in oxygen levels, cellular energy balance (for example, the ATP/ADP ratio) and the activity of certain transcription factors can all stimulate increased expression of ETC-related genes (Van Waveren and Moraes 2008; Fuhrmann and Brüne 2017; Chicherin et al. 2019; Srivastava et al. 2020; Ősz et al. 2025).

We examined the expression of three genes involved in the ETC. The *nd1* gene encodes a subunit of Complex I, while *bcs1* and *rip1* are associated with Complex III. Bcs1 is an AAA-ATPase that utilises ATP hydrolysis to facilitate protein translocation. In contrast, Rip1 functions as an assembly factor containing a [2Fe-2S] iron-sulphur cluster, essential for its role in accepting and transferring electrons to cytochrome c1 (Wirth et al. 2016; Ndi et al. 2018; Hou et al. 2023).

It has been reported that *nd1* expression can increase under oxidative stress as a compensatory mechanism and its upregulation together with the *bcs1* and *rip1* genes has been associated with cell cycle progression, resistance to antifungal drugs, enhanced ETC efficiency via increased ATP synthesis and adaptation to hypoxic conditions (Korde et al. 2011; Hou et al. 2023; Lin et al. 2024; Yang et al. 2024). These three genes are essential for mitochondrial integrity and function (Lin et al. 2024). Their similar upregulation patterns observed in hybrids d(NF418 × T1, n) and d(NS423 × T1, t) may result from selective pressures preserving co-evolved mitonuclear interactions, which leads to improved ETC functioning, ATP production and increased oxidative stress.

In the hybrid d(NS423 × T1, t), the upregulation of apoptotic genes such as *aif1*, encoding an apoptosis-inducing factor (Aif1, ID 1113817) and *mca1* (ID 1114660) which encodes a type I metacaspase involved in cell death (Sevrioukova 2011; Mukherjee et al. 2017), would suggest the occurrence of Programmed Cell Death (PCD) as a result of oxidative stress arising after mating of two parental lines with mismatches in their transcriptional regulatory mechanisms (Modjtahedi et al. 2006; Krantic et al. 2007; Dinamarco et al. 2010) (Suppl. material 1: fig. S7A–D).

Cox4 is an essential subunit of Complex IV in the mitochondrial electron transport chain, facilitating the transfer of electrons from cytochrome c to oxygen, which results in water formation. Elevated Cox4 levels can enhance mitochondrial efficiency and support overall cell health (Bikas et al. 2020; Čunátová et al. 2020; Yu et al. 2024). Cox5b, another subunit of Complex IV, operates alongside Cox4 to maintain respiratory function. Its increased expression can elevate Complex IV activity and ATP production, though its impact may vary depending on cell type and environmental conditions. In yeast, the *cox5b* gene and its counterpart *cox5a* are reciprocally regulated in an oxygen-dependent manner (Burke et al. 1997; Liu and Barrientos 2013).

In this study, we observed that both genes were upregulated in response to high oxidative stress (*cox4*) and hypoxia (*cox5b*) in slow-growing bred lines and their hybrids. The upregulation of *cox4* in slow-growing bred lines may result from elevated oxidative stress caused by disrupted co-evolved mitonuclear interactions, which also impairs ETC function in the slow-growing hybrid strains d(NS423 × T1, n) and d(NS426 × T1, n/t). In these hybrids, the transgressive expression (overdominance) of *cox4* highlights epistatic genetic incompatibilities between interacting genomes, resulting in failure to properly rewire transcriptional mechanisms. There are two main isoforms of *cox4*: *cox4-1*, the predominant form and *cox4-2*, which is typically upregulated under conditions of hypoxia and oxidative stress (Douiev et al. 2021). In the hybrid strain d(NS423 × T1, t), the downregulation of the *cox4* gene suggests that the (t) mitochondrial type may help to alleviate oxidative stress and support more efficient ETC function. It was reported that, in both mammals and yeast, the induction of hypoxia-responsive nuclear genes requires mitochondrial respiration (Dagsgaard et al. 2001). In this context, cytochrome c oxidase acts as an oxygen sensor.

In yeast, it was shown that the gene pair *cox5a*/*cyc1* encodes the normoxic isoforms (Cox5a and iso1-Cyc), while *cox5b*/*cyc7* encodes the hypoxic isoforms (Cox5b and iso2-Cyc), whose expression is influenced by ROS production (Liu and Barrientos 2013). In this study, the expression pattern of *cox5b* in both parental and hybrid strains mirrored that of the *cox4* gene. Its upregulation may serve as a marker of oxygen deprivation in slow-growing hybrids, with the exception of d(NS423 × T1, t), in which *cox5b* was downregulated. This observation suggests that the (t) mitochondrial type in this strain may contribute to restoring *cox5b* expression to levels typical of normoxic, fast-growing hybrids.

In summary, these findings highlight the influence of mitochondrial type on the transgressive gene expression and heterosis observed for *cox4* and *cox5b* in the d(NS423 × T1, n) hybrid.

In eukaryotic organisms, the primary antioxidant defence system includes *sod1*, *cat* and *gpx* genes encoding enzymes which play essential and non-redundant roles in cellular antioxidant defence, particularly in neutralising the superoxide anion radical (O_2_•-), reactive species continuously generated as a by-product of normal metabolism, especially during mitochondrial energy production. The expression levels of these enzymes respond to the degree of oxidative stress (Ighodaro and Akinloye 2018; Roy et al. 2023; Jomova et al. 2024).

Broadly expressed and primarily localised in the cytoplasm, Sod1 is constitutively present at basal levels, with its expression further regulated according to oxidative stress and cellular demands (Ighodaro and Akinloye 2018; Roy et al. 2023; Jomova et al. 2024). The observed upregulation of *sod1* in the slow-growing bred line mNS423 (n) likely reflects oxidative stress resulting from mitonuclear incompatibilities. In the hybrid strain d(NS423 × T1, t) the upregulation of *sod1* could indicate the influence of the (t) mitochondrial type in improving ETC functioning.

Cat acts as a defence mechanism against oxidative stress, helping to maintain redox balance and preventing apoptosis (Bai and Cederbaum 2003; Jena et al. 2023). The upregulation of *cat* is often part of an adaptive response allowing cells to tolerate elevated H_2_O_2_ levels (Ighodaro and Akinloye 2018; Roy et al. 2023; Jomova et al. 2024).

Gpx plays a central role in the cellular antioxidant defence system, particularly under hypoxic conditions reducing both H_2_O_2_ and hydroperoxides, to protect different cell components from oxidative damage. While Cat acts as a primary defence against moderate H_2_O_2_ accumulation, Gpx is essential for managing high concentrations of H_2_O_2_ and organic hydroperoxides (Masaki et al. 1998; Thu et al. 2010; Lei et al. 2023).

In this study, the expression patterns of *cat* and *gpx* genes distinguished the two types of parental bred lines and their hybrids. The *cat* expression was upregulated in fast-growing bred lines, whereas *gpx* expression was elevated in slow-growing lines. This differential expression likely reflects their distinct roles in antioxidant defence: Cat primarily detoxifies H_2_O_2_, while Gpx targets both H_2_O_2_ and organic hydroperoxides, as well as the specific redox environments in which these lines operate.

Notably, some hybrid strains exhibited a mitochondria-dependent shift in the expression of these genes (transgressive inheritance), with opposing expression patterns depending on the mitochondrial background. Similar results were reported by Wang et al. (Wang et al. 2024) in hybrids between channel catfish females and blue catfish males. However, in that case, mitochondrial type did not influence heterosis. Instead, environment-dependent heterosis and transgressive gene expression explained the observed hybrid variation under different environmental conditions.

The remarkable performance of the d(NS423 × T1, t) hybrid strain across multiple qualitative and quantitative traits was consistently observed when compared to other fast- and slow-growing hybrids under industrial cultivation and mushroom production conditions. These findings further underscore the importance of mitonuclear interactions in determining key agronomic traits such as spawn colonisation efficiency, substrate weight loss and total mushroom yield. The performance of this hybrid was comparable to that of fast-growing hybrids, suggesting that its superior productivity may be attributed to the presence of the (t) mitochondrial type. This mitochondrial background likely contributes to reducing oxidative stress and hypoxia levels, thereby enhancing growth rate and ultimately improving mushroom production. An alternative explanation for the performance of the d(NS423 × T1, t) hybrid could be its origin from a cross between two genetically divergent lines: the slow-growing bred line mNS423 (n) (recessive) and the mT1 (t) strain (dominant). In light of this and according to Bar-Zvi et al. (2017), the performance of the newly-formed hybrid heterokaryon could be interpreted either as the masking of deleterious parental mutations or as the disruption of growth-limiting regulatory systems.

Despite the outperformance of the hybrid d(NS423 × T1, t), laccase genes were not involved in spawn colonisation and substrate degradation. The expression of the *lacc4* gene allowed the identification of fast and slow-growing bred lines and their hybrids. This was not the case when *Coprinopsis cinerea* was co-cultivated with *Gongronella* sp. (Liu et al. 2022). This experimental system showed the upregulation of the *lacc9* gene as a result of a defence mechanism triggered against oxidative stress resulting from fungal-fungal interactions.

We hypothesise that the genomic incompatibilities arising from hybridisation between divergent parental lines could be similar to the co-cultivation scenario described above, though, in our study, oxidative stress and hypoxia responses in hybrids were primarily managed through the upregulation of *sod1*, *cat* and *gpx* genes, rather than *lacc4*. The enhanced growth rate of the d(NS423 × T1, t) hybrid compared with d(NS423 × T1, n) could be attributed to the upregulation of the *fet3* gene, which encodes a ferroxidase enzyme previously identified in *P. ostreatus* as *lacc5* (Castanera et al. 2013). This gene was described in *Oryza sativa* (Ai et al. 2020) as being involved in growth and development, thus supporting our findings.

As previously described, the *yfh1* and *fet3* genes function together as components of the high-affinity iron transport system, which plays a critical role in maintaining iron homeostasis and managing oxidative stress. In yeast, it has been reported that cells lacking the *yfh1* gene expression, showed upregulation of *fet3* and other iron uptake genes (Radisky et al. 1999). In the hybrid d(NS423 × T1, t), the observed upregulation of *yfh1* expression may suggest a partial restoration of iron balance and improved mitochondrial respiratory efficiency influenced by the (t) mitochondrial genome. This finding is supported by the upregulation of the *fzo1* gene (ID 1058073, encoding a GTPase protein also known as mitofusin) (Rapaport et al. 1998) and the *cld1* gene (ID 1063846, encoding a cardiolipin, a phospholipid involved in several mitochondrial functions such as energy production, stabilisation of mitochondrial proteins and preservation of mitochondrial membrane structure and dynamics) (Su and Dowhan 2006) (Suppl. material 1: fig. S7E–H). These genes ensure proper mitochondrial morphology and function and are consistent with the expression profiles of the ETC and detoxification genes described in this hybrid, which may ultimately contribute to increased expression of quantitative traits associated with mushroom production.

## ﻿Conclusions

The results of this work provide clues that demonstrate hybrid breakdown (outbreeding depression) in *P. ostreatus*, as evidenced by both quantitative and qualitative traits in advanced generations and hybrids, which exhibit transgressive inheritance and mitochondria-dependent heterosis. These outcomes appear to stem from genetic incompatibilities arising from the disruption of co-evolved mitonuclear interactions, established in hybrids following the mating of divergent parental strains. This disruption triggers enhanced oxidative stress, leading to the expression of genes related to the ETC, detoxification and PCD.

Our data analysis supports that, in *P. ostreatus*, the quantitative decline in fitness, ultimately leading to strain degeneration, can be avoided if the two F_1_ parental strains are maintained in long-term storage and the hybrid is remade shortly before use in commercial practice, mirroring what has been described for the production of hybrids in maize (Sumalini et al. 2018).

Currently, we are screening a set of putative bred lines for inclusion in new heterotic hybrids. However, our study may be limited by the availability of genomic and transcriptomic data of compatible parental lines that exhibit good general and specific combining abilities and tolerance to diverse substrates and temperatures for use in the breeding programme.

## ﻿Additional information

### Use of AI

During the preparation of this work, the authors used ChatGPT to assist with revising the English syntax and orthography. After utilizing this tool, the authors thoroughly reviewed and edited the content as necessary and take full responsibility for the published article’s content.

### Adherence to national and international regulations

All the fungal strains used in this study have been legally obtained, respecting the Convention on Biological Diversity (Rio Convention).

### Author ORCIDs

Edurne Garde https://orcid.org/0000-0002-5224-8257

Gumer Pérez https://orcid.org/0000-0003-2153-1962

Idoia Jiménez https://orcid.org/0000-0002-4460-3263

María Isabel Calvo https://orcid.org/0000-0002-3159-0336

Antonio G. Pisabarro https://orcid.org/0000-0001-6987-5794

Lucía Ramírez https://orcid.org/0000-0002-0023-4240

### Data availability

Data will be made available on request.

## Supplementary materials

Supplementary material 1 Supplementary information Data type docx Explanation note Contains the list of primers used in this paper, the amplification of nuclear and mitochondrial molecular markers, growth rate data of parental and hybrid strains at different temperatures and on different carbon sources, images of parental and hybrids strains, TLC images of compounds identified in hybrid fruiting bodies, gene expression, mode of inheritance and heterosis values. https://binary.pensoft.net/file/1454640This dataset is made available under the Open Database License (http://opendatacommons.org/licenses/odbl/1.0/). The Open Database License (ODbL) is a license agreement intended to allow users to freely share, modify, and use this Dataset while maintaining this same freedom for others, provided that the original source and author(s) are credited.Edurne Garde, Gumer Pérez, Idoia Jiménez, María Isabel Calvo, Antonio G. Pisabarro, Lucía Ramírez
