# Supplementary material for: ﻿Asymmetric mitonuclear interactions trigger transgressive inheritance and mitochondria-dependent heterosis in hybrids of the model system Pleurotus ostreatus
Source: IMA Fungus. 2025 Oct 31;16:e165520. doi: 10.3897/imafungus.16.165520 (PMC12595512; doi:10.3897/imafungus.16.165520)
Supplement: Supplementary material 1 — Supplementary information [file imafungus-16-e165520-s001.docx]

**Table S1**: Primer sequences used to amplify nuclear genes, reference genes, and nuclear and mitochondrial molecular markers.

| **PRIMER SEQUENCES (5’ to 3’) USED IN THIS STUDY** | |
| --- | --- |
| MOLECULAR MARKERS AMPLIFICATION | |
| F: GGCGGCAGAAATGACAGAAA | NUCLEAR: chromosome VII region |
| R: CAAAATGAAATCCGCACCGC |  |
| F: TGCTGGTATGATTGGTACTGC | MITOCHONDRIAL: Cox1 |
| R: AATGCCATATCAGGAGCTCC |  |
| ANALYSIS OF GENE EXPRESSION | |
| F: TGGTTCCGAGCTACTTTACCT | Nd1 (-) |
| R: AAGCAACACAAACAGGTAAAAGA |  |
| F: TGGACTCTTGAACGCATTGG | Bcs1 (ID 1105500) |
| R: GTTCCCGTGCTTGGATCTTG |  |
| F: CCCATAGGCCTTGTTTCCAA | Rip1 (ID 1088400) |
| R: CACATCCGAAGCGTCTTTGA |  |
| F: CTTCCAACGAGCGTGCTATC | Cox4 (ID 1087668) |
| R: GTGCTGTACGAAACCCTTCC |  |
| F: GCCGACTCTCATGACCTTCT | Cox5b (ID 1094413) |
| R: CTAATGGTGGCCATGGTCTT |  |
| F: - | Sod1* (ID 1113505) |
| R: - |  |
| F: TACTCCGGCCATACGTTCAA | Cat (ID 1090819) |
| R: GGTTCTGAACAGCTTCTCCG |  |
| F: TAAAGGAGAGGAAGGCTGGC | Gpx (ID 1090305) |
| R: ACGTTCCCCTCCTTGTCAAT |  |
| F: - | Lacc4** (ID 1077328) |
| R: - |  |
| F: - | Fet3 (Lacc5)** (ID 1094975) |
| R: - |  |
| F: GCCCAATTCCCCTTTCGGA | Yfh1 (ID 1095974) |
| R: GCCATATCTGCTTGTTGGGT |  |
| F: - | Fzo1* (ID 1058073) |
| R: - |  |
| F: TGGAGTCCGTTTCAGGTTGT | Cld1 (ID 1063846) |
| R: GCATATCCCTGGTCTCCTCC |  |
| F: - | Aif1* (ID 1113817) |
| R: - |  |
| F: AAAGATGAGGCACCCAAGGA | Mca1 (ID 1114660) |
| R: ACGGTTCTTGCACTGACCTA |  |
| REFERENCE GENES USED FOR THE ANALYSIS OF GENE EXPRESSION | |
| F: - | Sar1* (ID 1052294) |
| R: - |  |
| F: CGACGCCGCTGGTAAGAC | Sar4 (ID 1089490) |
| R: CAGACGGTGAATGAGATGTTCT |  |
| F: TCGTATCCTCAGCGACAGAG | Sar5 (ID 1089130) |
| R: TCTCTGTTACTTCGGCTGGG |  |

* See Pérez et al. (Pérez et al. 2021).

** See Castanera et al. (Castanera et al. 2013).


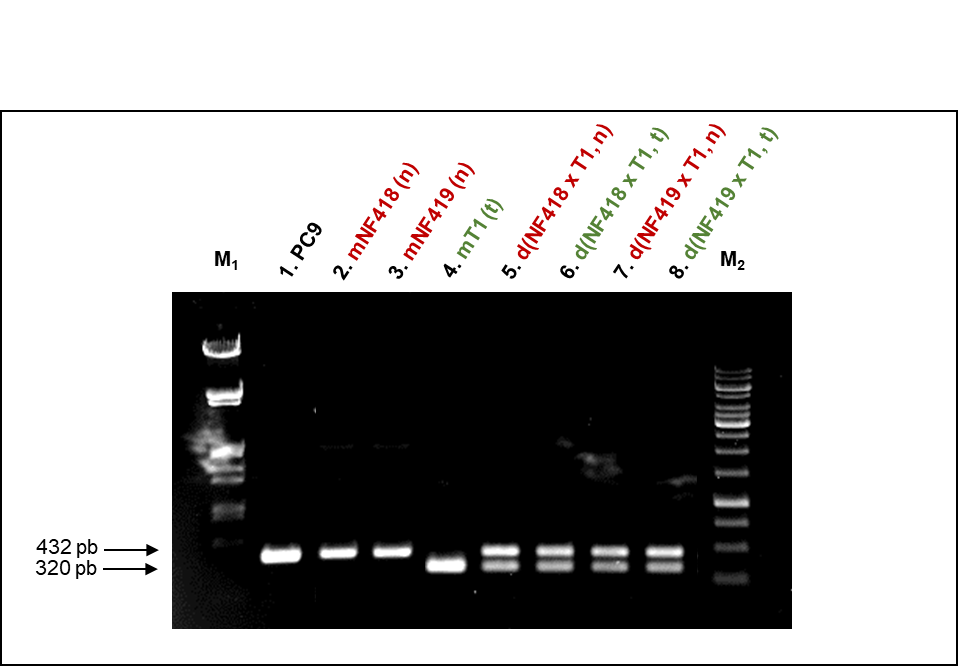


**Figure S1**. Identification of fast-growing hybrid strains. The PC9 protoclon and fast-growing bred lines carry a 0.4 kb fragment (lanes 1, 2, 3) whereas a 0.3 kb band was amplified in mT1 (t) strain (lane 4). Fast-growing hybrids carried both fragments (lanes 5, 6, 7 and 8). Lambda DNA/EcoRI + HindIII (Thermo Fisher Scientific, MA, USA) digested and the GeneRuler 1 kb DNA Ladder (Thermo Fisher Scientific, MA, USA) were used as molecular markers M_1_ and M_2_, respectively.


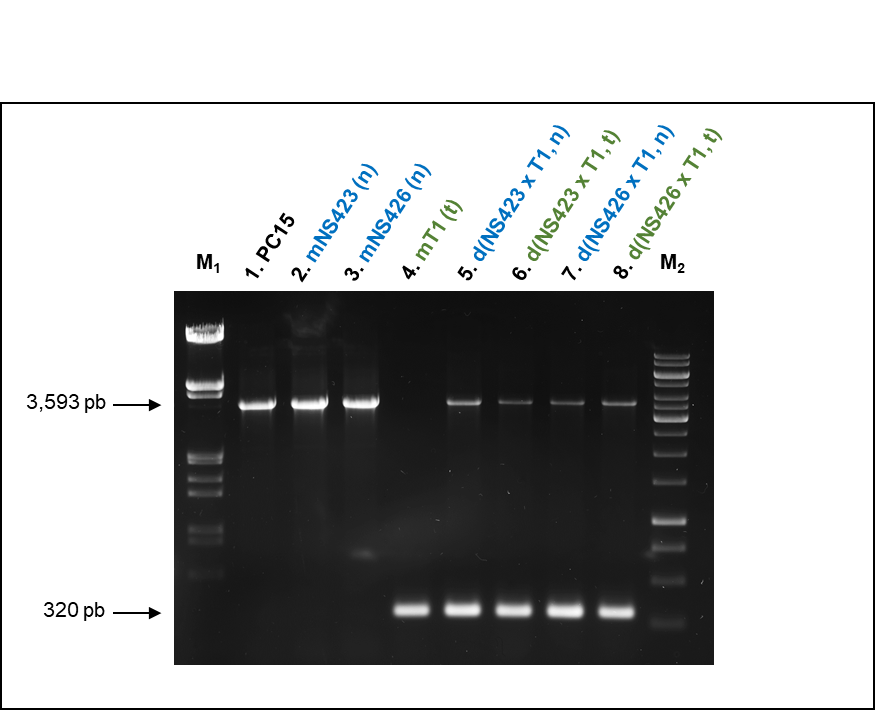


**Figure S2**. Identification of slow-growing hybrid strains. The PC15 protoclon and slow-growing bred lines carry a 3.6 kb fragment (lanes 1, 2 and 3), whereas a 0.3 kb band was amplified in mT1 (t) strain (lane 4). Slow-growing hybrids carried both fragments (lanes 5, 6, 7 and 8). Lambda DNA/EcoRI + HindIII (Thermo Fisher Scientific, MA, USA) digested and the GeneRuler 1 kb DNA Ladder (Thermo Fisher Scientific, MA, USA) were used as molecular markers M_1_ and M_2_, respectively.


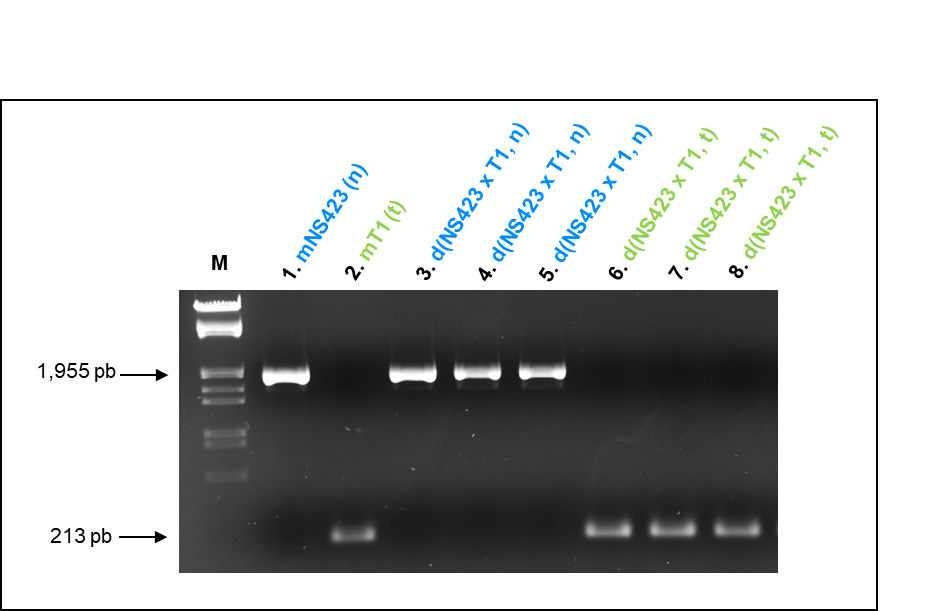


**Figure S3**. Identification of mitochondrial DNA inheritance. The mitochondrial DNA of the dN001 (n) strain carries a 1.96 kb fragment, while a 0.2 kb fragment is present in the mitochondrial DNA of the mT1 (t) strain. Lambda DNA/EcoRI + HindIII (Thermo Fisher Scientific, MA, USA) digested was used as M molecular marker.

**Table S2**. Growth rate values (mm/day) corresponding to the advanced F_2_, F_3_ and F_4_ fast and slow-growing lines. Growth rate values represent the average growth rate of 30 fast- and slow-growing monokaryons ± Standard Deviation (SD).

| **Offspring** | **Growth rate (mm/day)** |
| --- | --- |
| Fast-growing line |  |
| F2 | 2.04 ± 0.48 |
| F3 | 3.26 ± 0.59 |
| F4 | 4.07 ± 0.38 |
| Slow-growing line |  |
| S2 | 1.14 ± 0.27 |
| S3 | 1.19 ± 0.48 |
| S4 | 1.12 ± 0.47 |


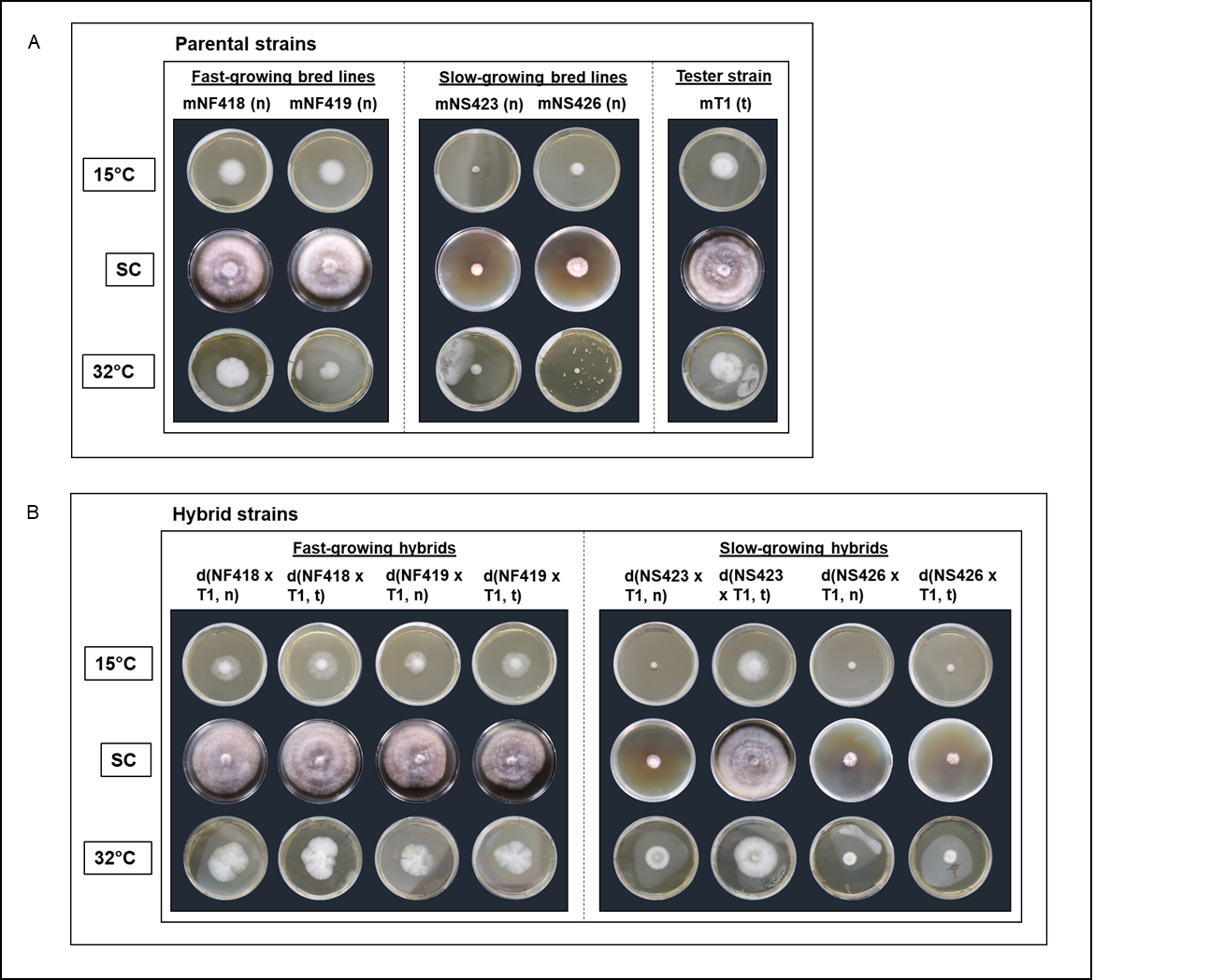


**Figure S4**. Mycelial growth rate on the seventh day of cultivation under SC and at different temperatures for the parental strains (A) and their hybrids (B).

**Table S3.1**. Growth rate of parental strains under SC, at different temperatures and grown on different carbon sources. Values (mm/day) represent the mean of three replicates ± SD.

| **Culture conditions** | **Growth rate of parental strains** | | | | |
| --- | --- | --- | --- | --- | --- |
|  | **mNF418 (n)** | **mNF419 (n)** | **mNS423 (n)** | **mNS426 (n)** | **mT1 (t)** |
| **15°C** | 2.24 ± 0.13 | 2.23 ± 0.03 | 0.74 ± 0.01 | 1.38 ± 0.06 | 2.27 ± 0.04 |
| **SC** | 4.25 ±0.27 | 4.10 ±0.17 | 0.89 ± 0.03 | 1.74 ± 0.06 | 5.07 ± 0.39 |
| **32°C** | 2.42 ± 0.06 | 1.55 ± 0.27 | 1.00 ± 0.01 | 0.55 ± 0.03 | 2.16 ± 0.40 |
| **MSM + Glu** | 4.17 ± 0.11 | 3.84 ± 0.14 | 0.95 ± 0.02 | 1.69 ± 0.17 | 4.35 ± 0.30 |
| **MSM + Suc** | 4.50 ± 0.14 | 4.13 ± 0.11 | 1.00 ± 0.00 | 2.75 ± 0.03 | 4.19 ± 0.18 |
| **MSM + Gly** | 3.65 ± 0.05 | 3.18 ± 0.07 | 0.93 ± 0.03 | 2.32 ± 0.03 | 1.71 ± 0.02 |

(n) is the mitochondrial type inherited from the dN001 (n) strain.

(t) is the mitochondrial type inherited from the dT009 (t) strain.

**Table S3.2**. Growth rate of hybrid strains under SC, at different temperatures and grown on different carbon sources. Values (mm/day) represent the mean of three replicates ± SD.

| **Culture conditions** | **Growth rate of hybrid strains** | | | | | | | |
| --- | --- | --- | --- | --- | --- | --- | --- | --- |
|  | **d(NF418 x T1, n)** | **d(NF418 x T1, t)** | **d(NF419 x T1, n)** | **d(NF419 x T1, t)** | **d(NS423 x T1, n)** | **d(NS423 x T1, t)** | **d(NS426 x T1, n)** | **d(NS426 x T1, t)** |
| **15°C** | 2.78 ± 0.41 | 2.54 ± 0.05 | 1.81 ± 0.11 | 2.12 ± 0.38 | 0.65 ± 0.00 | 2.39 ± 0.39 | 0.90 ± 0.08 | 0.74 ± 0.06 |
| **SC** | 5.09 ±0.18 | 5.11 ±0.22 | 3.43 ± 0.18 | 4.04 ± 0.48 | 1.15 ± 0.10 | 4.18 ± 0.50 | 1.89 ± 0.29 | 1.58 ± 0.07 |
| **32°C** | 2.66 ± 0.40 | 2.97 ± 0.24 | 2.34 ± 0.17 | 2.30 ± 0.15 | 1.70 ± 0.13 | 2.78 ± 0.27 | 1.71 ± 0.16 | 1.60 ± 0.22 |
| **MSM + Glu** | 4.44 ± 0.19 | 4.16 ± 0.23 | 4.32 ± 0.18 | 4.46 ± 0.12 | 0.94 ± 0.07 | 3.50 ± 0.50 | 1.22 ± 0.19 | 1.04 ± 0.10 |
| **MSM + Suc** | 4.89 ± 0.10 | 5.08 ± 0.28 | 4.63 ± 0.15 | 4.42 ± 0.09 | 1.01 ± 0.11 | 3.70 ± 0.52 | 1.58 ± 0.23 | 1.56 ± 0.03 |
| **MSM +**  **Gly** | 3.29 ± 0.01 | 3.32 ± 0.10 | 2.69 ± 0.04 | 3.01 ± 0.09 | 1.02 ± 0.28 | 2.80 ± 0.30 | 1.27 ± 0.19 | 1.30 ± 0.20 |

(n) is the mitochondrial type inherited from the dN001 (n) strain.

(t) is the mitochondrial type inherited from the dT009 (t) strain.


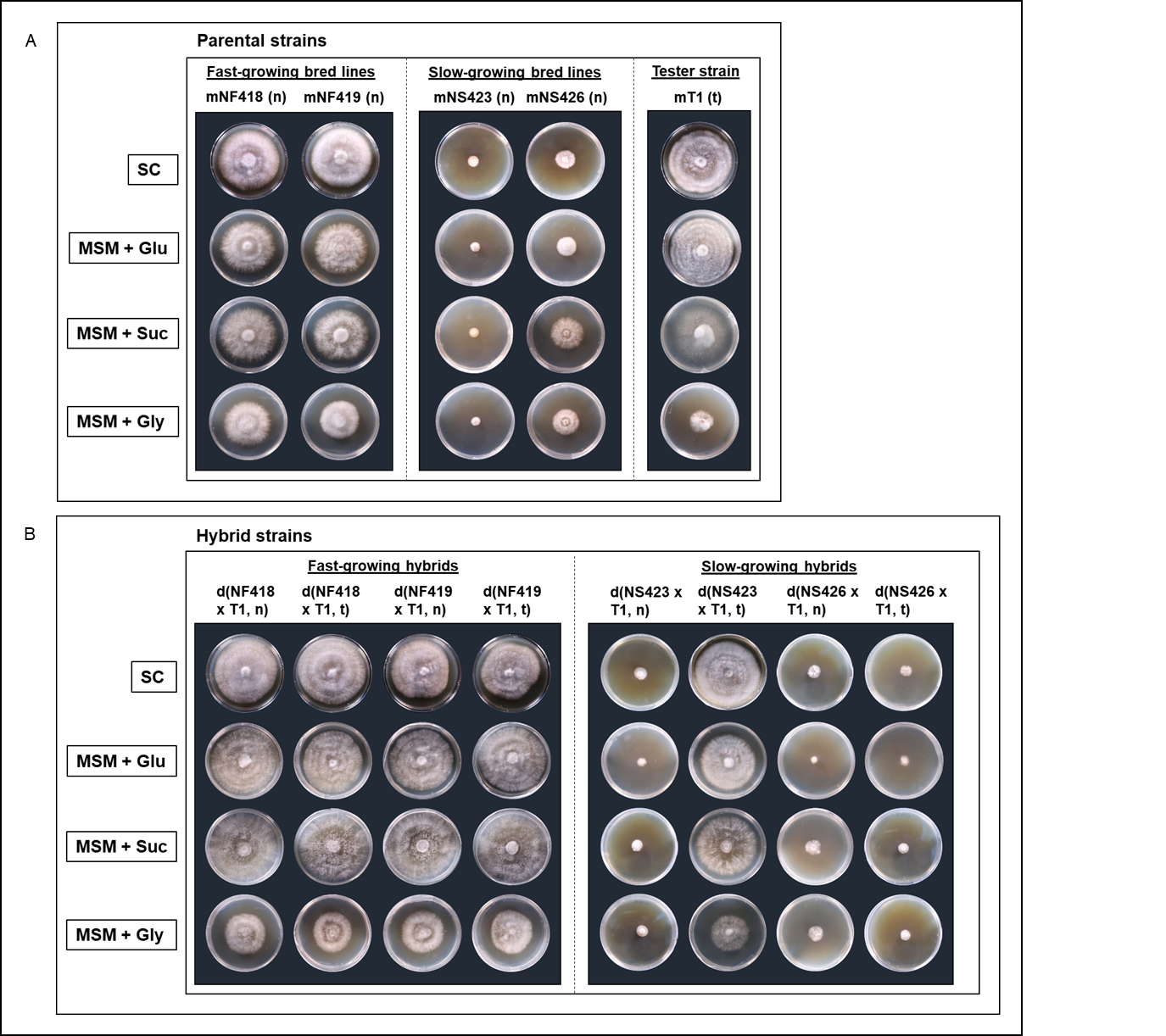


**Figure S5.**  Mycelial growth rate on the seventh day of cultivation under SC and on different carbon sources: (A) parental strains and (B) hybrid strains.


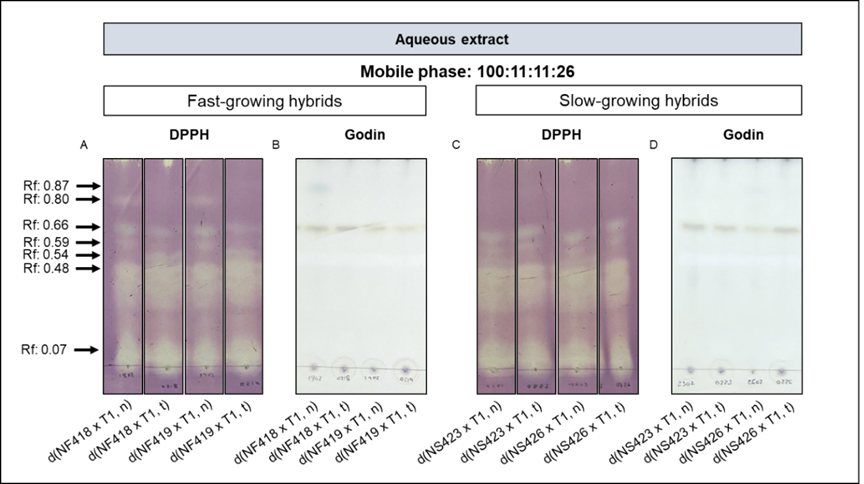


**Figure S6**. TLC plate profiles obtained from extracts of hybrid strain fruiting bodies. Plates show molecular compounds stained with DPPH (A and C) and Godin’s reagent (B and D).

A B

a

a

b

a

a

a

a

a

b

b

a

ab

ab

C D

b

b

c

b

a

a

a

a

c

bc

b

a

a

E F

b

b

c

b

ab

ab

ab

a

c

ac

b

a

a

G H

a

a

b

a

a

a

a

a

a

a

b

c

d

a

a

a

a

a

a

a

b

**Figure S7**. Relative expression level of genes related to apoptosis (A), (B), (C) (D), mitochondrial fusion, division and integrity (E), (F), (G), (H), in parental and hybrid strains.

**Table S4**. **MPH1.** Growth rate **M**id-**P**arent **H**eterosis (**MPH**) values of fast- and slow-growing hybrid strains under SC and at different temperatures.

|  | **Growth rate (mm/day)** | | | | | **Heterosis** | |
| --- | --- | --- | --- | --- | --- | --- | --- |
|  | **Parental strains** | | **Hybrid strains** | |  | **MPH** | |
|  | mNF418 (n) | mT1 (t) | d(NF418 x T1, n) | d(NF418 x T1, t) | **Mid-parent value (m)** | d(NF418 x T1, n) | d(NF418 x T1, t) |
| **15°C** | 2.24 | 2.27 | 2.78 | 2.54 | 2.26 | 0.23 | 0.13 |
| **SC** | 4.25 | 5.07 | 5.09 | 5.11 | 4.66 | 0.09 | 0.10 |
| **32°C** | 2.42 | 2.16 | 2.66 | 2.97 | 2.29 | **0.16** | **0.30** |
|  | mNF419 (n) | mT1 (t) | d(NF419 x T1, n) | d(NF419 x T1, t) | **Mid-parent value (m)** | d(NF419 x T1, n) | d(NF419 x T1, t) |
| **15°C** | 2.23 | 2.27 | 1.81 | 2.12 | 2.25 | -0.20 | -0.06 |
| **SC** | 4.10 | 5.07 | 3.43 | 4.04 | 4.59 | -0.25 | -0.12 |
| **32°C** | 1.55 | 2.16 | 2.34 | 2.30 | 1.86 | **0.26** | **0.24** |
|  | mNS423 (n) | mT1 (t) | d(NS423 x T1, n) | d(NS423 x T1, t) | **Mid-parent value (m)** | d(NS423 x T1, n) | d(NS423 x T1, t) |
| **15°C** | 0.74 | 2.27 | 0.65 | 2.39 | 1.51 | -0.57 | **0.59** |
| **SC** | 0.89 | 5.07 | 1.15 | 4.18 | 2.98 | -0.61 | **0.40** |
| **32°C** | 1.00 | 2.16 | 1.70 | 2.78 | 1.58 | 0.08 | **0.76** |
|  | mNS426 (n) | mT1 (t) | d(NS426 x T1, n) | d(NS426 x T1, t) | **Mid-parent value (m)** | d(NS426 x T1, n) | d(NS426 x T1, t) |
| **15°C** | 1.38 | 2.27 | 0.90 | 0.74 | 1.83 | -0.51 | -0.59 |
| **SC** | 1.74 | 5.07 | 1.89 | 1.58 | 3.41 | -0.44 | -0.54 |
| **32°C** | 0.55 | 2.16 | 1.71 | 1.60 | 1.36 | 0.26 | 0.18 |

Light yellow: values represent the growth rate of the parental strains in mm/day under SC at different temperatures.

Light green: values represent the growth rate of the hybrid strains in mm/day under SC at different temperatures.

(n) is the mitochondrial type inherited from the dN001 (n) strain.

(t) is the mitochondrial type inherited from the dT009 (t) strain.

(m) and MPH were calculated as it was described in Materials and methods.

**Table S5**. **MPH2.** Growth rate **M**id-**P**arent **H**eterosis (**MPH**) values of fast- and slow-growing hybrid strains under SC and on different carbon sources.

|  | **Growth rate (mm/day)** | | | | | **Heterosis** | |
| --- | --- | --- | --- | --- | --- | --- | --- |
|  | **Parental strains** | | **Hybrid strains** | |  | **MPH** | |
|  | mNF418 (n) | mT1 (t) | d(NF418 x T1, n) | d(NF418 x T1, t) | **Mid-parent value (m)** | d(NF418 x T1, n) | d(NF418 x T1, t) |
| **SC** | 4.25 | 5.07 | 5.09 | 5.11 | 4.66 | 0.09 | 0.10 |
| **MSM + Glu** | 4.17 | 4.35 | 4.44 | 4.16 | 4.26 | 0.04 | -0.02 |
| **MSM + Suc** | 4.50 | 4.19 | 4.89 | 5.08 | 4.35 | 0.13 | 0.17 |
| **MSM + Gly** | 3.65 | 1.71 | 3.29 | 3.32 | 2.68 | **0.23** | **0.24** |
|  | mNF419 (n) | mT1 (t) | d(NF419 x T1, n) | d(NF419 x T1, t) | **Mid-parent value (m)** | d(NF419 x T1, n) | d(NF419 x T1, t) |
| **SC** | 4.10 | 5.07 | 3.43 | 4.04 | 4.59 | -0.25 | -0.12 |
| **MSM + Glu** | 3.84 | 4.35 | 4.32 | 4.46 | 4.10 | 0.05 | 0.09 |
| **MSM + Suc** | 4.13 | 4.19 | 4.63 | 4.42 | 4.16 | 0.11 | 0.06 |
| **MSM + Gly** | 3.18 | 1.71 | 2.69 | 3.01 | 2.45 | **0.10** | **0.23** |
|  | mNS423 (n) | mT1 (t) | d(NS423 x T1, n) | d(NS423 x T1, t) | **Mid-parent value (m)** | d(NS423 x T1, n) | d(NS423 x T1, t) |
| **SC** | 0.89 | 5.07 | 1.15 | 4.18 | 2.98 | -0.61 | **0.40** |
| **MSM + Glu** | 0.95 | 4.35 | 0.94 | 3.50 | 2.65 | -0.65 | **0.32** |
| **MSM + Suc** | 1.00 | 4.19 | 1.01 | 3.70 | 2.60 | -0.61 | **0.43** |
| **MSM + Gly** | 0.93 | 1.71 | 1.02 | 2.80 | 1.32 | -0.23 | **1.12** |
|  | mNS426 (n) | mT1 (t) | d(NS426 x T1, n) | d(NS426 x T1, t) | **Mid-parent value (m)** | d(NS426 x T1, n) | d(NS426 x T1, t) |
| **SC** | 1.74 | 5.07 | 1.89 | 1.58 | 3.41 | -0.44 | -0.54 |
| **MSM + Glu** | 1.69 | 4.35 | 1.22 | 1.04 | 3.02 | -0.60 | -0.66 |
| **MSM + Suc** | 2.75 | 4.19 | 1.58 | 1.56 | 3.47 | -0.54 | -0.55 |
| **MSM + Gly** | 2.32 | 1.71 | 1.27 | 1.30 | 2.02 | -0.37 | -0.35 |

Light yellow: values represent the growth rate of the parental strains in mm/day under SC and on different carbon sources.

Light green: values represent the growth rate of the hybrid strains in mm/day under SC and on different carbon sources.

(n) is the mitochondrial type inherited from the dN001 (n) strain.

(t) is the mitochondrial type inherited from the dT009 (t) strain.

(m) and MPH were calculated as it was described in Materials and methods.

**Table S6**. **MPH3.** **M**id-**P**arent **H**eterosis (**MPH**) values for *nd1* gene expression in fast- and slow-growing hybrid strains.

|  | **Relative expression** | | | | | **Heterosis** | |
| --- | --- | --- | --- | --- | --- | --- | --- |
|  | **Parental strains** | | **Hybrid strains** | |  | **MPH** | |
|  | mNF418 (n) | mT1 (t) | d(NF418 x T1, n) | d(NF418 x T1, t) | **Mid-parent value (m)** | d(NF418 x T1, n) | d(NF418 x T1, t) |
| ***nd1*** | 1.11 | 13.19 | 9.06 | 1.23 | 7.15 | 0.27 | -0.83 |
|  | mNF419 (n) | mT1 (t) | d(NF419 x T1, n) | d(NF419 x T1, t) | **Mid-parent value (m)** | d(NF419 x T1, n) | d(NF419 x T1, t) |
|  | 0.66 | 13.19 | 1.26 | 1.60 | 6.92 | -0.82 | -0.77 |
|  | mNS423 (n) | mT1 (t) | d(NS423 x T1, n) | d(NS423 x T1, t) | **Mid-parent value (m)** | d(NS423 x T1, n) | d(NS423 x T1, t) |
|  | 1.15 | 13.19 | 3.23 | 14.72 | 7.17 | -0.55 | **1.05** |
|  | mNS426 (n) | mT1 (t) | d(NS426 x T1, n) | d(NS426 x T1, t) | **Mid-parent value (m)** | d(NS426 x T1, n) | d(NS426 x T1, t) |
|  | 0.39 | 13.19 | 2.36 | 0.69 | 6.79 | -0.65 | -0.90 |

Light yellow: values represent the average of three replicates of nd1 relative gene expression in the parental strains.

Light green: values represent the average of three replicates of *nd1* relative gene expression of the hybrid strains.

(n) is the mitochondrial type inherited from the dN001 (n) strain.

(t) is the mitochondrial type inherited from the dT009 (t) strain.

(m) and MPH were calculated as it was described in Materials and methods.

**Table S7. MPH4.** **M**id-**P**arent **H**eterosis (**MPH**) values for *bcs1* gene expression in fast- and slow-growing hybrid strains.

|  | **Relative expression** | | | | | **Heterosis** | |
| --- | --- | --- | --- | --- | --- | --- | --- |
|  | **Parental strains** | | **Hybrid strains** | |  | **MPH** | |
|  | mNF418 (n) | mT1 (t) | d(NF418 x T1, n) | d(NF418 x T1, t) | **Mid-parent value (m)** | d(NF418 x T1, n) | d(NF418 x T1, t) |
| ***bcs1*** | 0.04 | 0.16 | 0.15 | 0.05 | 0.10 | **0.47** | -0.52 |
|  | mNF419 (n) | mT1 (t) | d(NF419 x T1, n) | d(NF419 x T1, t) | **Mid-parent value (m)** | d(NF419 x T1, n) | d(NF419 x T1, t) |
|  | 0.07 | 0.16 | 0.05 | 0.06 | 0.12 | -0.60 | -0.44 |
|  | mNS423 (n) | mT1 (t) | d(NS423 x T1, n) | d(NS423 x T1, t) | **Mid-parent value (m)** | d(NS423 x T1, n) | d(NS423 x T1, t) |
|  | 0.05 | 0.16 | 0.06 | 0.38 | 0.11 | -0.44 | **2.52** |
|  | mNS426 (n) | mT1 (t) | d(NS426 x T1, n) | d(NS426 x T1, t) | **Mid-parent value (m)** | d(NS426 x T1, n) | d(NS426 x T1, t) |
|  | 0.06 | 0.16 | 0.03 | 0.04 | 0.11 | -0.71 | -0.61 |

Light yellow: values represent the average of three replicates of bcs1 relative gene expression in the parental strains.

Light green: values represent the average of three replicates of *bcs1* relative gene expression of the hybrid strains.

(n) is the mitochondrial type inherited from the dN001 (n) strain.

(t) is the mitochondrial type inherited from the dT009 (t) strain.

(m) and MPH were calculated as it was described in Materials and methods.

**Table S8**. **MPH5.** **M**id-**P**arent **H**eterosis (**MPH**) values for *rip1* gene expression in fast- and slow-growing hybrid strains.

|  | **Relative expression** | | | | | **Heterosis** | |
| --- | --- | --- | --- | --- | --- | --- | --- |
|  | **Parental strains** | | **Hybrid strains** | |  | **MPH** | |
|  | mNF418 (n) | mT1 (t) | d(NF418 x T1, n) | d(NF418 x T1, t) | **Mid-parent value (m)** | d(NF418 x T1, n) | d(NF418 x T1, t) |
| ***rip1*** | 0.01 | 0.00 | 0.01 | 0.01 | 0.01 | **1.73** | 0.23 |
|  | mNF419 (n) | mT1 (t) | d(NF419 x T1, n) | d(NF419 x T1, t) | **Mid-parent value (m)** | d(NF419 x T1, n) | d(NF419 x T1, t) |
|  | 0.01 | 0.00 | 0.01 | 0.01 | 0.01 | 0.38 | 0.59 |
|  | mNS423 (n) | mT1 (t) | d(NS423 x T1, n) | d(NS423 x T1, t) | **Mid-parent value (m)** | d(NS423 x T1, n) | d(NS423 x T1, t) |
|  | 0.00 | 0.00 | 0.00 | 0.02 | 0.00 | 0.22 | **5.33** |
|  | mNS426 (n) | mT1 (t) | d(NS426 x T1, n) | d(NS426 x T1, t) | **Mid-parent value (m)** | d(NS426 x T1, n) | d(NS426 x T1, t) |
|  | 0.03 | 0.00 | 0.01 | 0.00 | 0.01 | -0.62 | -0.80 |

Light yellow: values represent the average of three replicates of rip1 relative gene expression in the parental strains.

Light green: values represent the average of three replicates of *rip1* relative gene expression of the hybrid strains.

(n) is the mitochondrial type inherited from the dN001 (n) strain.

(t) is the mitochondrial type inherited from the dT009 (t) strain.

(m) and MPH were calculated as it was described in Materials and methods.

**Table S9**. **MPH6.** **M**id-**P**arent **H**eterosis (**MPH**) values for *cox4* gene expression in fast- and slow-growing hybrid strains.

|  | **Relative expression** | | | | | **Heterosis** | |
| --- | --- | --- | --- | --- | --- | --- | --- |
|  | **Parental strains** | | **Hybrid strains** | |  | **MPH** | |
|  | mNF418 (n) | mT1 (t) | d(NF418 x T1, n) | d(NF418 x T1, t) | **Mid-parent value (m)** | d(NF418 x T1, n) | d(NF418 x T1, t) |
| ***cox4*** | 0.13 | 0.57 | 0.53 | 0.27 | 0.35 | 0.50 | -0.24 |
|  | mNF419 (n) | mT1 (t) | d(NF419 x T1, n) | d(NF419 x T1, t) | **Mid-parent value (m)** | d(NF419 x T1, n) | d(NF419 x T1, t) |
|  | 0.15 | 0.57 | 0.29 | 0.30 | 0.36 | -0.20 | -0.17 |
|  | mNS423 (n) | mT1 (t) | d(NS423 x T1, n) | d(NS423 x T1, t) | **Mid-parent value (m)** | d(NS423 x T1, n) | d(NS423 x T1, t) |
|  | 0.52 | 0.57 | 3.83 | 0.92 | 0.55 | **6.02** | 0.68 |
|  | mNS426 (n) | mT1 (t) | d(NS426 x T1, n) | d(NS426 x T1, t) | **Mid-parent value (m)** | d(NS426 x T1, n) | d(NS426 x T1, t) |
|  | 0.96 | 0.57 | 2.45 | 1.26 | 0.77 | 2.18 | 0.64 |

Light yellow: values represent the average of three replicates of cox4 relative gene expression in the parental strains.

Light green: values represent the average of three replicates of *cox4* relative gene expression of the hybrid strains.

(n) is the mitochondrial type inherited from the dN001 (n) strain.

(t) is the mitochondrial type inherited from the dT009 (t) strain.

(m) and MPH were calculated as it was described in Materials and methods.

**Table S10**. **MPH7.** **M**id-**P**arent **H**eterosis (**MPH**) values for *cox5b* gene expression in fast- and slow-growing hybrid strains.

|  | **Relative expression** | | | | | **Heterosis** | |
| --- | --- | --- | --- | --- | --- | --- | --- |
|  | **Parental strains** | | **Hybrid strains** | |  | **MPH** | |
|  | mNF418 (n) | mT1 (t) | d(NF418 x T1, n) | d(NF418 x T1, t) | **Mid-parent value (m)** | d(NF418 x T1, n) | d(NF418 x T1, t) |
| ***cox5b*** | 0.16 | 0.00 | 0.29 | 0.17 | 0.08 | 2.54 | 1.09 |
|  | mNF419 (n) | mT1 (t) | d(NF419 x T1, n) | d(NF419 x T1, t) | **Mid-parent value (m)** | d(NF419 x T1, n) | d(NF419 x T1, t) |
|  | 0.20 | 0.00 | 0.18 | 0.25 | 0.10 | 0.82 | 1.52 |
|  | mNS423 (n) | mT1 (t) | d(NS423 x T1, n) | d(NS423 x T1, t) | **Mid-parent value (m)** | d(NS423 x T1, n) | d(NS423 x T1, t) |
|  | 1.27 | 0.00 | 2.19 | 0.42 | 0.63 | **2.45** | -0.33 |
|  | mNS426 (n) | mT1 (t) | d(NS426 x T1, n) | d(NS426 x T1, t) | **Mid-parent value (m)** | d(NS426 x T1, n) | d(NS426 x T1, t) |
|  | 1.41 | 0.00 | 1.33 | 0.97 | 0.71 | 0.88 | 0.37 |

Light yellow: values represent the average of three replicates of cox5b relative gene expression in the parental strains.

Light green: values represent the average of three replicates of *cox5b* relative gene expression of the hybrid strains.

(n) is the mitochondrial type inherited from the dN001 (n) strain.

(t) is the mitochondrial type inherited from the dT009 (t) strain.

(m) and MPH were calculated as it was described in Materials and methods.

**Table S11**. **MPH8.** **M**id-**P**arent **H**eterosis (**MPH**) values for *sod1* gene expression in fast- and slow-growing hybrid strains.

|  | **Relative expression** | | | | | **Heterosis** | |
| --- | --- | --- | --- | --- | --- | --- | --- |
|  | **Parental strains** | | **Hybrid strains** | |  | **MPH** | |
|  | mNF418 (n) | mT1 (t) | d(NF418 x T1, n) | d(NF418 x T1, t) | **Mid-parent value (m)** | d(NF418 x T1, n) | d(NF418 x T1, t) |
| ***sod1*** | 0.01 | 0.01 | 0.07 | 0.02 | 0.01 | **8.04** | **1.09** |
|  | mNF419 (n) | mT1 (t) | d(NF419 x T1, n) | d(NF419 x T1, t) | **Mid-parent value (m)** | d(NF419 x T1, n) | d(NF419 x T1, t) |
|  | 0.01 | 0.01 | 0.01 | 0.02 | 0.01 | **0.63** | **1.73** |
|  | mNS423 (n) | mT1 (t) | d(NS423 x T1, n) | d(NS423 x T1, t) | **Mid-parent value (m)** | d(NS423 x T1, n) | d(NS423 x T1, t) |
|  | 0.06 | 0.01 | 0.09 | 0.21 | 0.03 | **1.49** | **4.94** |
|  | mNS426 (n) | mT1 (t) | d(NS426 x T1, n) | d(NS426 x T1, t) | **Mid-parent value (m)** | d(NS426 x T1, n) | d(NS426 x T1, t) |
|  | 0.01 | 0.01 | 0.02 | 0.01 | 0.01 | **1.20** | **0.35** |

Light yellow: values represent the average of three replicates of sod1 relative gene expression in the parental strains.

Light green: values represent the average of three replicates of *sod1* relative gene expression of the hybrid strains.

(n) is the mitochondrial type inherited from the dN001 (n) strain.

(t) is the mitochondrial type inherited from the dT009 (t) strain.

(m) and MPH were calculated as it was described in Materials and methods.

**Table S12**. **MPH9.** **M**id-**P**arent **H**eterosis (**MPH**) values for *cat* gene expression in fast- and slow-growing hybrid strains.

|  | **Relative expression** | | | | | **Heterosis** | |
| --- | --- | --- | --- | --- | --- | --- | --- |
|  | **Parental strains** | | **Hybrid strains** | |  | **MPH** | |
|  | mNF418 (n) | mT1 (t) | d(NF418 x T1, n) | d(NF418 x T1, t) | **Mid-parent value (m)** | d(NF418 x T1, n) | d(NF418 x T1, t) |
| ***cat*** | 0.79 | 0.71 | 0.52 | 1.38 | 0.75 | -0.31 | **0.84** |
|  | mNF419 (n) | mT1 (t) | d(NF419 x T1, n) | d(NF419 x T1, t) | **Mid-parent value (m)** | d(NF419 x T1, n) | d(NF419 x T1, t) |
|  | 0.82 | 0.71 | 0.89 | 0.84 | 0.77 | 0.15 | 0.09 |
|  | mNS423 (n) | mT1 (t) | d(NS423 x T1, n) | d(NS423 x T1, t) | **Mid-parent value (m)** | d(NS423 x T1, n) | d(NS423 x T1, t) |
|  | 0.18 | 0.71 | 0.11 | 0.34 | 0.45 | -0.76 | -0.24 |
|  | mNS426 (n) | mT1 (t) | d(NS426 x T1, n) | d(NS426 x T1, t) | **Mid-parent value (m)** | d(NS426 x T1, n) | d(NS426 x T1, t) |
|  | 0.08 | 0.71 | 0.06 | 0.09 | 0.40 | -0.86 | -0.78 |

Light yellow: values represent the average of three replicates of cat relative gene expression in the parental strains.

Light green: values represent the average of three replicates of *cat* relative gene expression of the hybrid strains.

(n) is the mitochondrial type inherited from the dN001 (n) strain.

(t) is the mitochondrial type inherited from the dT009 (t) strain.

(m) and MPH were calculated as it was described in Materials and methods.

**Table S13**. **MPH10.** **M**id-**P**arent **H**eterosis (**MPH**) values for *gpx* gene expression in fast- and slow-growing hybrid strains.

|  | **Relative expression** | | | | | **Heterosis** | |
| --- | --- | --- | --- | --- | --- | --- | --- |
|  | **Parental strains** | | **Hybrid strains** | |  | **MPH** | |
|  | mNF418 (n) | mT1 (t) | d(NF418 x T1, n) | d(NF418 x T1, t) | **Mid-parent value (m)** | d(NF418 x T1, n) | d(NF418 x T1, t) |
| ***gpx*** | 0.08 | 0.12 | 0.15 | 0.07 | 0.10 | **0.50** | -0.34 |
|  | mNF419 (n) | mT1 (t) | d(NF419 x T1, n) | d(NF419 x T1, t) | **Mid-parent value (m)** | d(NF419 x T1, n) | d(NF419 x T1, t) |
|  | 0.08 | 0.12 | 0.07 | 0.08 | 0.10 | -0.31 | -0.21 |
|  | mNS423 (n) | mT1 (t) | d(NS423 x T1, n) | d(NS423 x T1, t) | **Mid-parent value (m)** | d(NS423 x T1, n) | d(NS423 x T1, t) |
|  | 0.19 | 0.12 | 0.43 | 0.33 | 0.16 | **1.71** | **1.07** |
|  | mNS426 (n) | mT1 (t) | d(NS426 x T1, n) | d(NS426 x T1, t) | **Mid-parent value (m)** | d(NS426 x T1, n) | d(NS426 x T1, t) |
|  | 0.25 | 0.12 | 0.29 | 0.24 | 0.19 | 0.54 | 0.29 |

Light yellow: values represent the average of three replicates of gpx relative gene expression in the parental strains.

Light green: values represent the average of three replicates of *gpx* relative gene expression of the hybrid strains.

(n) is the mitochondrial type inherited from the dN001 (n) strain.

(t) is the mitochondrial type inherited from the dT009 (t) strain.

(m) and MPH were calculated as it was described in Materials and methods.

**Table S1**. **MI1.** **M**ode of **I**nheritance (**MI**) of the *nd1* gene in fast- and slow-growing hybrids.

| **Relative expression of *nd1*** | | | | | **Additive genetic deviation (a)** | | **Dominant genetic deviation (d)** | | **d/a** | | | | **Mode of inheritance** | | | |
| --- | --- | --- | --- | --- | --- | --- | --- | --- | --- | --- | --- | --- | --- | --- | --- | --- |
| **Parental strains** | | **Hybrid strains** | |  |  |  |  |  | **d(NF418 x T1, n)** | **d(NF418 x T1, t)** | **d(NF418 x T1, n)** | **d(NF418 x T1, t)** | **d(NF418 x T1, n)** | **d(NF418 x T1, t)** | **d(NF418 x T1, n)** | **d(NF418 x T1, t)** |
| **mNF418 (n)** | **mT1 (t)** | **d(NF418 x T1, n)** | **d(NF418 x T1, t)** | **Mid-parent value (m)** | **mNF418 (n)** | **mT1 (t)** | **d(NF418 x T1, n)** | **d(NF418 x T1, t)** | **mNF418 (n)** | | **mT1 (t)** | | **mNF418 (n)** | | **mT1 (t)** | |
| 1.11 | 13.19 | 9.06 | 1.23 | 7.15 | -6.04 | 6.04 | 1.91 | -5.92 | -0.32 | 0.98 | 0.32 | -0.98 | PD | D | **PD** | D |
|  |  |  |  |  |  |  |  |  | **d(NF419 x T1, n)** | **d(NF419 x T1, t)** | **d(NF419 x T1, n)** | **d(NF419 x T1, t)** | **d(NF419 x T1, n)** | **d(NF419 x T1, t)** | **d(NF419 x T1, n)** | **d(NF419 x T1, t)** |
| **mNF419 (n)** | **mT1 (t)** | **d(NF419 x T1, n)** | **d(NF419 x T1, t)** | **Mid-parent value (m)** | **mNF419 (n)** | **mT1 (t)** | **d(NF419 x T1, n)** | **d(NF419 x T1, t)** | **mNF419 (n)** | | **mT1 (t)** | | **mNF419 (n)** | | **mT1 (t)** | |
| 0.66 | 13.19 | 1.26 | 1.60 | 6.92 | -6.27 | 6.27 | -5.67 | -5.32 | 0.90 | 0.85 | -0.90 | -0.85 | D | PD | D | PD |
|  |  |  |  |  |  |  |  |  | **d(NS423 x T1, n)** | **d(NS423 x T1, t)** | **d(NS423 x T1, n)** | **d(NS423 x T1, t)** | **d(NS423 x T1, n)** | **d(NS423 x T1, t)** | **d(NS423 x T1, n)** | **d(NS423 x T1, t)** |
| **mNS423 (n)** | **mT1 (t)** | **d(NS423 x T1, n)** | **d(NS423 x T1, t)** | **Mid-parent value (m)** | **mNS423 (n)** | **mT1 (t)** | **d(NS423 x T1, n)** | **d(NS423 x T1, t)** | **mNS423 (n)** | | **mT1 (t)** | | **mNS423 (n)** | | **mT1 (t)** | |
| 1.15 | 13.19 | 3.23 | 14.72 | 7.17 | -6.02 | 6.02 | -3.94 | 7.55 | 0.66 | -1.25 | -0.66 | 1.25 | PD | UD | PD | **OD** |
|  |  |  |  |  |  |  |  |  | **d(NS426 x T1, n)** | **d(NS426 x T1, t)** | **d(NS426 x T1, n)** | **d(NS426 x T1, t)** | **d(NS426 x T1, n)** | **d(NS426 x T1, t)** | **d(NS426 x T1, n)** | **d(NS426 x T1, t)** |
| **mNS426 (n)** | **mT1 (t)** | **d(NS426 x T1, n)** | **d(NS426 x T1, t)** | **Mid-parent value (m)** | **mNS426 (n)** | **mT1 (t)** | **d(NS426 x T1, n)** | **d(NS426 x T1, t)** | **mNS426 (n)** | | **mT1 (t)** | | **mNS426 (n)** | | **mT1 (t)** | |
| 0.39 | 13.19 | 2.36 | 0.69 | 6.79 | -6.40 | 6.40 | -4.43 | -6.10 | 0.69 | 0.95 | -0.69 | -0.95 | PD | D | PD | D |

Light blue: values represent the average of three replicates of nd1 relative gene expression in the parental strains.

Light red: values represent the average of three replicates of *nd1* relative gene expression of the hybrid strains.

(n) is the mitochondrial type inherited from the dN001 (n) strain.

(t) is the mitochondrial type inherited from the dT009 (t) strain.

(m), (a), (d) and (d/a) were calculated as it was described in Materials and methods.

C: Codominance; PD: Partial Dominance; D: Full Dominance; OD: Overdominance; UD: Underdominance

**Table S2. MI2.** **M**ode of **I**nheritance (**MI**) of the *bcs1* gene in fast- and slow-growing hybrids.

| **Relative expression of *bcs1*** | | | | | **Additive genetic deviation (a)** | | **Dominant genetic deviation (d)** | | **d/a** | | | | **Mode of inheritance** | | | |
| --- | --- | --- | --- | --- | --- | --- | --- | --- | --- | --- | --- | --- | --- | --- | --- | --- |
| **Parental strains** | | **Hybrid strains** | |  |  |  |  |  | **d(NF418 x T1, n)** | **d(NF418 x T1, t)** | **d(NF418 x T1, n)** | **d(NF418 x T1, t)** | **d(NF418 x T1, n)** | **d(NF418 x T1, t)** | **d(NF418 x T1, n)** | **d(NF418 x T1, t)** |
| **mNF418 (n)** | **mT1 (t)** | **d(NF418 x T1, n)** | **d(NF418 x T1, t)** | **Mid-parent value (m)** | **mNF418 (n)** | **mT1 (t)** | **d(NF418 x T1, n)** | **d(NF418 x T1, t)** | **mNF418 (n)** | | **mT1 (t)** | | **mNF418 (n)** | | **mT1 (t)** | |
| 0.04 | 0.16 | 0.15 | 0.05 | 0.10 | -0.06 | 0.06 | 0.05 | -0.05 | -0.73 | 0.81 | 0.73 | -0.81 | PD | PD | **PD** | PD |
|  |  |  |  |  |  |  |  |  | **d(NF419 x T1, n)** | **d(NF419 x T1, t)** | **d(NF419 x T1, n)** | **d(NF419 x T1, t)** | **d(NF419 x T1, n)** | **d(NF419 x T1, t)** | **d(NF419 x T1, n)** | **d(NF419 x T1, t)** |
| **mNF419 (n)** | **mT1 (t)** | **d(NF419 x T1, n)** | **d(NF419 x T1, t)** | **Mid-parent value (m)** | **mNF419 (n)** | **mT1 (t)** | **d(NF419 x T1, n)** | **d(NF419 x T1, t)** | **mNF419 (n)** | | **mT1 (t)** | | **mNF419 (n)** | | **mT1 (t)** | |
| 0.07 | 0.16 | 0.05 | 0.06 | 0.12 | -0.05 | 0.05 | -0.07 | -0.05 | 1.46 | 1.09 | -1.46 | -1.09 | OD | D | UD | D |
|  |  |  |  |  |  |  |  |  | **d(NS423 x T1, n)** | **d(NS423 x T1, t)** | **d(NS423 x T1, n)** | **d(NS423 x T1, t)** | **d(NS423 x T1, n)** | **d(NS423 x T1, t)** | **d(NS423 x T1, n)** | **d(NS423 x T1, t)** |
| **mNS423 (n)** | **mT1 (t)** | **d(NS423 x T1, n)** | **d(NS423 x T1, t)** | **Mid-parent value (m)** | **mNS423 (n)** | **mT1 (t)** | **d(NS423 x T1, n)** | **d(NS423 x T1, t)** | **mNS423 (n)** | | **mT1 (t)** | | **mNS423 (n)** | | **mT1 (t)** | |
| 0.05 | 0.16 | 0.06 | 0.38 | 0.11 | -0.06 | 0.06 | -0.05 | 0.27 | 0.84 | -4.83 | -0.84 | 4.83 | PD | UD | PD | **OD** |
|  |  |  |  |  |  |  |  |  | **d(NS426 x T1, n)** | **d(NS426 x T1, t)** | **d(NS426 x T1, n)** | **d(NS426 x T1, t)** | **d(NS426 x T1, n)** | **d(NS426 x T1, t)** | **d(NS426 x T1, n)** | **d(NS426 x T1, t)** |
| **mNS426 (n)** | **mT1 (t)** | **d(NS426 x T1, n)** | **d(NS426 x T1, t)** | **Mid-parent value (m)** | **mNS426 (n)** | **mT1 (t)** | **d(NS426 x T1, n)** | **d(NS426 x T1, t)** | **mNS426 (n)** | | **mT1 (t)** | | **mNS426 (n)** | | **mT1 (t)** | |
| 0.06 | 0.16 | 0.03 | 0.04 | 0.11 | -0.05 | 0.05 | -0.08 | -0.07 | 1.61 | 1.39 | -1.61 | -1.39 | OD | OD | UD | UD |

Light blue: values represent the average of three replicates of bcs1 relative gene expression in the parental strains.

Light red: values represent the average of three replicates of *bcs1* relative gene expression of the hybrid strains.

(n) is the mitochondrial type inherited from the dN001 (n) strain.

(t) is the mitochondrial type inherited from the dT009 (t) strain.

(m), (a), (d) and (d/a) were calculated as it was described in Materials and methods.

C: Codominance; PD: Partial Dominance; D: Full Dominance; OD: Overdominance; UD: Underdominance

**Table S3. MI3.** **M**ode of **I**nheritance (**MI**) of the *rip1* gene in fast- and slow-growing hybrids.

| **Relative expression of *rip1*** | | | | | **Additive genetic deviation (a)** | | **Dominant genetic deviation (d)** | | **d/a** | | | | **Mode of inheritance** | | | |
| --- | --- | --- | --- | --- | --- | --- | --- | --- | --- | --- | --- | --- | --- | --- | --- | --- |
| **Parental strains** | | **Hybrid strains** | |  |  |  |  |  | **d(NF418 x T1, n)** | **d(NF418 x T1, t)** | **d(NF418 x T1, n)** | **d(NF418 x T1, t)** | **d(NF418 x T1, n)** | **d(NF418 x T1, t)** | **d(NF418 x T1, n)** | **d(NF418 x T1, t)** |
| **mNF418 (n)** | **mT1 (t)** | **d(NF418 x T1, n)** | **d(NF418 x T1, t)** | **Mid-parent value (m)** | **mNF418 (n)** | **mT1 (t)** | **d(NF418 x T1, n)** | **d(NF418 x T1, t)** | **mNF418 (n)** | | **mT1 (t)** | | **mNF418 (n)** | | **mT1 (t)** | |
| 0.01 | 0.00 | 0.01 | 0.01 | 0.01 | 0.00 | 0.00 | 0.01 | 0.00 | 3.05 | 0.40 | -3.05 | -0.40 | **OD** | PD | UD | PD |
|  |  |  |  |  |  |  |  |  | **d(NF419 x T1, n)** | **d(NF419 x T1, t)** | **d(NF419 x T1, n)** | **d(NF419 x T1, t)** | **d(NF419 x T1, n)** | **d(NF419 x T1, t)** | **d(NF419 x T1, n)** | **d(NF419 x T1, t)** |
| **mNF419 (n)** | **mT1 (t)** | **d(NF419 x T1, n)** | **d(NF419 x T1, t)** | **Mid-parent value (m)** | **mNF419 (n)** | **mT1 (t)** | **d(NF419 x T1, n)** | **d(NF419 x T1, t)** | **mNF419 (n)** | | **mT1 (t)** | | **mNF419 (n)** | | **mT1 (t)** | |
| 0.01 | 0.00 | 0.01 | 0.01 | 0.01 | 0.00 | 0.00 | 0.00 | 0.00 | 0.62 | 0.96 | -0.62 | -0.96 | PD | D | PD | D |
|  |  |  |  |  |  |  |  |  | **d(NS423 x T1, n)** | **d(NS423 x T1, t)** | **d(NS423 x T1, n)** | **d(NS423 x T1, t)** | **d(NS423 x T1, n)** | **d(NS423 x T1, t)** | **d(NS423 x T1, n)** | **d(NS423 x T1, t)** |
| **mNS423 (n)** | **mT1 (t)** | **d(NS423 x T1, n)** | **d(NS423 x T1, t)** | **Mid-parent value (m)** | **mNS423 (n)** | **mT1 (t)** | **d(NS423 x T1, n)** | **d(NS423 x T1, t)** | **mNS423 (n)** | | **mT1 (t)** | | **mNS423 (n)** | | **mT1 (t)** | |
| 0.00 | 0.00 | 0.00 | 0.02 | 0.00 | 0.00 | 0.00 | 0.00 | 0.02 | 0.97 | 23.15 | -0.97 | -23.15 | D | **OD** | D | UD |
|  |  |  |  |  |  |  |  |  | **d(NS426 x T1, n)** | **d(NS426 x T1, t)** | **d(NS426 x T1, n)** | **d(NS426 x T1, t)** | **d(NS426 x T1, n)** | **d(NS426 x T1, t)** | **d(NS426 x T1, n)** | **d(NS426 x T1, t)** |
| **mNS426 (n)** | **mT1 (t)** | **d(NS426 x T1, n)** | **d(NS426 x T1, t)** | **Mid-parent value (m)** | **mNS426 (n)** | **mT1 (t)** | **d(NS426 x T1, n)** | **d(NS426 x T1, t)** | **mNS426 (n)** | | **mT1 (t)** | | **mNS426 (n)** | | **mT1 (t)** | |
| 0.03 | 0.00 | 0.01 | 0.00 | 0.01 | 0.01 | -0.01 | -0.01 | -0.01 | -0.73 | -0.95 | 0.73 | 0.95 | PD | D | PD | D |

Light blue: values represent the average of three replicates of rip1 relative gene expression in the parental strains.

Light red: values represent the average of three replicates of *rip1* relative gene expression of the hybrid strains.

(n) is the mitochondrial type inherited from the dN001 (n) strain.

(t) is the mitochondrial type inherited from the dT009 (t) strain.

(m), (a), (d) and (d/a) were calculated as it was described in Materials and methods.

C: Codominance; PD: Partial Dominance; D: Full Dominance; OD: Overdominance; UD: Underdominance

**Table S4**. **MI4.** **M**ode of **I**nheritance (**MI**) of the *cox4* gene in fast- and slow-growing hybrids.

| **Relative expression of *cox4*** | | | | | **Additive genetic deviation (a)** | | **Dominant genetic deviation (d)** | | **d/a** | | | | **Mode of inheritance** | | | |
| --- | --- | --- | --- | --- | --- | --- | --- | --- | --- | --- | --- | --- | --- | --- | --- | --- |
| **Parental strains** | | **Hybrid strains** | |  |  |  |  |  | **d(NF418 x T1, n)** | **d(NF418 x T1, t)** | **d(NF418 x T1, n)** | **d(NF418 x T1, t)** | **d(NF418 x T1, n)** | **d(NF418 x T1, t)** | **d(NF418 x T1, n)** | **d(NF418 x T1, t)** |
| **mNF418 (n)** | **mT1 (t)** | **d(NF418 x T1, n)** | **d(NF418 x T1, t)** | **Mid-parent value (m)** | **mNF418 (n)** | **mT1 (t)** | **d(NF418 x T1, n)** | **d(NF418 x T1, t)** | **mNF418 (n)** | | **mT1 (t)** | | **mNF418 (n)** | | **mT1 (t)** | |
| 0.13 | 0.57 | 0.53 | 0.27 | 0.35 | -0.22 | 0.22 | 0.18 | -0.08 | -0.79 | 0.37 | 0.79 | -0.37 | PD | PD | PD | PD |
|  |  |  |  |  |  |  |  |  | **d(NF419 x T1, n)** | **d(NF419 x T1, t)** | **d(NF419 x T1, n)** | **d(NF419 x T1, t)** | **d(NF419 x T1, n)** | **d(NF419 x T1, t)** | **d(NF419 x T1, n)** | **d(NF419 x T1, t)** |
| **mNF419 (n)** | **mT1 (t)** | **d(NF419 x T1, n)** | **d(NF419 x T1, t)** | **Mid-parent value (m)** | **mNF419 (n)** | **mT1 (t)** | **d(NF419 x T1, n)** | **d(NF419 x T1, t)** | **mNF419 (n)** | | **mT1 (t)** | | **mNF419 (n)** | | **mT1 (t)** | |
| 0.15 | 0.57 | 0.29 | 0.30 | 0.36 | -0.21 | 0.21 | -0.07 | -0.06 | 0.34 | 0.28 | -0.34 | -0.28 | PD | PD | PD | PD |
|  |  |  |  |  |  |  |  |  | **d(NS423 x T1, n)** | **d(NS423 x T1, t)** | **d(NS423 x T1, n)** | **d(NS423 x T1, t)** | **d(NS423 x T1, n)** | **d(NS423 x T1, t)** | **d(NS423 x T1, n)** | **d(NS423 x T1, t)** |
| **mNS423 (n)** | **mT1 (t)** | **d(NS423 x T1, n)** | **d(NS423 x T1, t)** | **Mid-parent value (m)** | **mNS423 (n)** | **mT1 (t)** | **d(NS423 x T1, n)** | **d(NS423 x T1, t)** | **mNS423 (n)** | | **mT1 (t)** | | **mNS423 (n)** | | **mT1 (t)** | |
| 0.52 | 0.57 | 3.83 | 0.92 | 0.55 | -0.03 | 0.03 | 3.28 | 0.37 | -114.32 | -12.89 | 114.32 | 12.89 | UD | UD | **OD** | **OD** |
|  |  |  |  |  |  |  |  |  | **d(NS426 x T1, n)** | **d(NS426 x T1, t)** | **d(NS426 x T1, n)** | **d(NS426 x T1, t)** | **d(NS426 x T1, n)** | **d(NS426 x T1, t)** | **d(NS426 x T1, n)** | **d(NS426 x T1, t)** |
| **mNS426 (n)** | **mT1 (t)** | **d(NS426 x T1, n)** | **d(NS426 x T1, t)** | **Mid-parent value (m)** | **mNS426 (n)** | **mT1 (t)** | **d(NS426 x T1, n)** | **d(NS426 x T1, t)** | **mNS426 (n)** | | **mT1 (t)** | | **mNS426 (n)** | | **mT1 (t)** | |
| 0.96 | 0.57 | 2.45 | 1.26 | 0.77 | 0.20 | -0.20 | 1.68 | 0.49 | 8.60 | 2.52 | -8.60 | -2.52 | **OD** | OD | UD | UD |

Light blue: values represent the average of three replicates of cox4 relative gene expression in the parental strains.

Light red: values represent the average of three replicates of *cox4* relative gene expression of the hybrid strains.

(n) is the mitochondrial type inherited from the dN001 (n) strain.

(t) is the mitochondrial type inherited from the dT009 (t) strain.

(m), (a), (d) and (d/a) were calculated as it was described in Materials and methods.

C: Codominance; PD: Partial Dominance; D: Full Dominance; OD: Overdominance; UD: Underdominance

**Table S5**. **MI5.** **M**ode of **I**nheritance (**MI**) of the *cox5b* gene in fast- and slow-growing hybrids.

| **Relative expression of *cox5b*** | | | | | **Additive genetic deviation (a)** | | **Dominant genetic deviation (d)** | | **d/a** | | | | **Mode of inheritance** | | | |
| --- | --- | --- | --- | --- | --- | --- | --- | --- | --- | --- | --- | --- | --- | --- | --- | --- |
| **Parental strains** | | **Hybrid strains** | |  |  |  |  |  | **d(NF418 x T1, n)** | **d(NF418 x T1, t)** | **d(NF418 x T1, n)** | **d(NF418 x T1, t)** | **d(NF418 x T1, n)** | **d(NF418 x T1, t)** | **d(NF418 x T1, n)** | **d(NF418 x T1, t)** |
| **mNF418 (n)** | **mT1 (t)** | **d(NF418 x T1, n)** | **d(NF418 x T1, t)** | **Mid-parent value (m)** | **mNF418 (n)** | **mT1 (t)** | **d(NF418 x T1, n)** | **d(NF418 x T1, t)** | **mNF418 (n)** | | **mT1 (t)** | | **mNF418 (n)** | | **mT1 (t)** | |
| 0.16 | 0.00 | 0.29 | 0.17 | 0.08 | 0.08 | -0.08 | 0.21 | 0.09 | 2.58 | 1.11 | -2.58 | -1.11 | OD | D | UD | D |
|  |  |  |  |  |  |  |  |  | **d(NF419 x T1, n)** | **d(NF419 x T1, t)** | **d(NF419 x T1, n)** | **d(NF419 x T1, t)** | **d(NF419 x T1, n)** | **d(NF419 x T1, t)** | **d(NF419 x T1, n)** | **d(NF419 x T1, t)** |
| **mNF419 (n)** | **mT1 (t)** | **d(NF419 x T1, n)** | **d(NF419 x T1, t)** | **Mid-parent value (m)** | **mNF419 (n)** | **mT1 (t)** | **d(NF419 x T1, n)** | **d(NF419 x T1, t)** | **mNF419 (n)** | | **mT1 (t)** | | **mNF419 (n)** | | **mT1 (t)** | |
| 0.20 | 0.00 | 0.18 | 0.25 | 0.10 | 0.10 | -0.10 | 0.08 | 0.15 | 0.83 | 1.53 | -0.83 | -1.53 | PD | OD | PD | UD |
|  |  |  |  |  |  |  |  |  | **d(NS423 x T1, n)** | **d(NS423 x T1, t)** | **d(NS423 x T1, n)** | **d(NS423 x T1, t)** | **d(NS423 x T1, n)** | **d(NS423 x T1, t)** | **d(NS423 x T1, n)** | **d(NS423 x T1, t)** |
| **mNS423 (n)** | **mT1 (t)** | **d(NS423 x T1, n)** | **d(NS423 x T1, t)** | **Mid-parent value (m)** | **mNS423 (n)** | **mT1 (t)** | **d(NS423 x T1, n)** | **d(NS423 x T1, t)** | **mNS423 (n)** | | **mT1 (t)** | | **mNS423 (n)** | | **mT1 (t)** | |
| 1.27 | 0.00 | 2.19 | 0.42 | 0.63 | 0.63 | -0.63 | 1.56 | -0.21 | 2.46 | -0.34 | -2.46 | 0.34 | **OD** | **PD** | UD | PD |
|  |  |  |  |  |  |  |  |  | **d(NS426 x T1, n)** | **d(NS426 x T1, t)** | **d(NS426 x T1, n)** | **d(NS426 x T1, t)** | **d(NS426 x T1, n)** | **d(NS426 x T1, t)** | **d(NS426 x T1, n)** | **d(NS426 x T1, t)** |
| **mNS426 (n)** | **mT1 (t)** | **d(NS426 x T1, n)** | **d(NS426 x T1, t)** | **Mid-parent value (m)** | **mNS426 (n)** | **mT1 (t)** | **d(NS426 x T1, n)** | **d(NS426 x T1, t)** | **mNS426 (n)** | | **mT1 (t)** | | **mNS426 (n)** | | **mT1 (t)** | |
| 1.41 | 0.00 | 1.33 | 0.97 | 0.71 | 0.71 | -0.71 | 0.63 | 0.26 | 0.89 | 0.37 | -0.89 | -0.37 | **PD** | PD | PD | PD |

Light blue: values represent the average of three replicates of cox5b relative gene expression in the parental strains.

Light red: values represent the average of three replicates of *cox5b* relative gene expression of the hybrid strains.

(n) is the mitochondrial type inherited from the dN001 (n) strain.

(t) is the mitochondrial type inherited from the dT009 (t) strain.

(m), (a), (d) and (d/a) were calculated as it was described in Materials and methods.

C: Codominance; PD: Partial Dominance; D: Full Dominance; OD: Overdominance; UD: Underdominance

**Table S6**. **MI6.** **M**ode of **I**nheritance (**MI**) of the *sod1* gene in fast- and slow-growing hybrids.

| **Relative expression of *sod1*** | | | | | **Additive genetic deviation (a)** | | **Dominant genetic deviation (d)** | | **d/a** | | | | **Mode of inheritance** | | | |
| --- | --- | --- | --- | --- | --- | --- | --- | --- | --- | --- | --- | --- | --- | --- | --- | --- |
| **Parental strains** | | **Hybrid strains** | |  |  |  |  |  | **d(NF418 x T1, n)** | **d(NF418 x T1, t)** | **d(NF418 x T1, n)** | **d(NF418 x T1, t)** | **d(NF418 x T1, n)** | **d(NF418 x T1, t)** | **d(NF418 x T1, n)** | **d(NF418 x T1, t)** |
| **mNF418 (n)** | **mT1 (t)** | **d(NF418 x T1, n)** | **d(NF418 x T1, t)** | **Mid-parent value (m)** | **mNF418 (n)** | **mT1 (t)** | **d(NF418 x T1, n)** | **d(NF418 x T1, t)** | **mNF418 (n)** | | **mT1 (t)** | | **mNF418 (n)** | | **mT1 (t)** | |
| 0.01 | 0.01 | 0.07 | 0.02 | 0.01 | 0.00 | 0.00 | 0.06 | 0.01 | 56.15 | 7.62 | -56.15 | -7.62 | **OD** | **OD** | UD | UD |
|  |  |  |  |  |  |  |  |  | **d(NF419 x T1, n)** | **d(NF419 x T1, t)** | **d(NF419 x T1, n)** | **d(NF419 x T1, t)** | **d(NF419 x T1, n)** | **d(NF419 x T1, t)** | **d(NF419 x T1, n)** | **d(NF419 x T1, t)** |
| **mNF419 (n)** | **mT1 (t)** | **d(NF419 x T1, n)** | **d(NF419 x T1, t)** | **Mid-parent value (m)** | **mNF419 (n)** | **mT1 (t)** | **d(NF419 x T1, n)** | **d(NF419 x T1, t)** | **mNF419 (n)** | | **mT1 (t)** | | **mNF419 (n)** | | **mT1 (t)** | |
| 0.01 | 0.01 | 0.01 | 0.02 | 0.01 | 0.00 | 0.00 | 0.00 | 0.01 | -32.09 | -88.18 | 32.09 | 88.18 | UD | UD | **OD** | **OD** |
|  |  |  |  |  |  |  |  |  | **d(NS423 x T1, n)** | **d(NS423 x T1, t)** | **d(NS423 x T1, n)** | **d(NS423 x T1, t)** | **d(NS423 x T1, n)** | **d(NS423 x T1, t)** | **d(NS423 x T1, n)** | **d(NS423 x T1, t)** |
| **mNS423 (n)** | **mT1 (t)** | **d(NS423 x T1, n)** | **d(NS423 x T1, t)** | **Mid-parent value (m)** | **mNS423 (n)** | **mT1 (t)** | **d(NS423 x T1, n)** | **d(NS423 x T1, t)** | **mNS423 (n)** | | **mT1 (t)** | | **mNS423 (n)** | | **mT1 (t)** | |
| 0.06 | 0.01 | 0.09 | 0.21 | 0.03 | 0.03 | -0.03 | 0.05 | 0.17 | 1.83 | 6.07 | -1.83 | -6.07 | **OD** | **OD** | UD | UD |
|  |  |  |  |  |  |  |  |  | **d(NS426 x T1, n)** | **d(NS426 x T1, t)** | **d(NS426 x T1, n)** | **d(NS426 x T1, t)** | **d(NS426 x T1, n)** | **d(NS426 x T1, t)** | **d(NS426 x T1, n)** | **d(NS426 x T1, t)** |
| **mNS426 (n)** | **mT1 (t)** | **d(NS426 x T1, n)** | **d(NS426 x T1, t)** | **Mid-parent value (m)** | **mNS426 (n)** | **mT1 (t)** | **d(NS426 x T1, n)** | **d(NS426 x T1, t)** | **mNS426 (n)** | | **mT1 (t)** | | **mNS426 (n)** | | **mT1 (t)** | |
| 0.01 | 0.01 | 0.02 | 0.01 | 0.01 | 0.00 | 0.00 | 0.01 | 0.00 | 5.55 | 1.64 | -5.55 | -1.64 | **OD** | **OD** | UD | UD |

Light blue: values represent the average of three replicates of sod1 relative gene expression in the parental strains.

Light red: values represent the average of three replicates of *sod1* relative gene expression of the hybrid strains.

(n) is the mitochondrial type inherited from the dN001 (n) strain.

(t) is the mitochondrial type inherited from the dT009 (t) strain.

(m), (a), (d) and (d/a) were calculated as it was described in Materials and methods.

C: Codominance; PD: Partial Dominance; D: Full Dominance; OD: Overdominance; UD: Underdominance

**Table S7**. **MI7.** **M**ode of **I**nheritance (**MI**) of the *cat* gene in fast- and slow-growing hybrids.

| **Relative expression of *cat*** | | | | | **Additive genetic deviation (a)** | | **Dominant genetic deviation (d)** | | **d/a** | | | | **Mode of inheritance** | | | |
| --- | --- | --- | --- | --- | --- | --- | --- | --- | --- | --- | --- | --- | --- | --- | --- | --- |
| **Parental strains** | | **Hybrid strains** | |  |  |  |  |  | **d(NF418 x T1, n)** | **d(NF418 x T1, t)** | **d(NF418 x T1, n)** | **d(NF418 x T1, t)** | **d(NF418 x T1, n)** | **d(NF418 x T1, t)** | **d(NF418 x T1, n)** | **d(NF418 x T1, t)** |
| **mNF418 (n)** | **mT1 (t)** | **d(NF418 x T1, n)** | **d(NF418 x T1, t)** | **Mid-parent value (m)** | **mNF418 (n)** | **mT1 (t)** | **d(NF418 x T1, n)** | **d(NF418 x T1, t)** | **mNF418 (n)** | | **mT1 (t)** | | **mNF418 (n)** | | **mT1 (t)** | |
| 0.79 | 0.71 | 0.52 | 1.38 | 0.75 | 0.04 | -0.04 | -0.23 | 0.63 | -5.76 | 15.44 | 5.76 | -15.44 | **UD** | **OD** | OD | UD |
|  |  |  |  |  |  |  |  |  | **d(NF419 x T1, n)** | **d(NF419 x T1, t)** | **d(NF419 x T1, n)** | **d(NF419 x T1, t)** | **d(NF419 x T1, n)** | **d(NF419 x T1, t)** | **d(NF419 x T1, n)** | **d(NF419 x T1, t)** |
| **mNF419 (n)** | **mT1 (t)** | **d(NF419 x T1, n)** | **d(NF419 x T1, t)** | **Mid-parent value (m)** | **mNF419 (n)** | **mT1 (t)** | **d(NF419 x T1, n)** | **d(NF419 x T1, t)** | **mNF419 (n)** | | **mT1 (t)** | | **mNF419 (n)** | | **mT1 (t)** | |
| 0.82 | 0.71 | 0.89 | 0.84 | 0.77 | 0.06 | -0.06 | 0.12 | 0.07 | 2.09 | 1.20 | -2.09 | -1.20 | OD | OD | UD | UD |
|  |  |  |  |  |  |  |  |  | **d(NS423 x T1, n)** | **d(NS423 x T1, t)** | **d(NS423 x T1, n)** | **d(NS423 x T1, t)** | **d(NS423 x T1, n)** | **d(NS423 x T1, t)** | **d(NS423 x T1, n)** | **d(NS423 x T1, t)** |
| **mNS423 (n)** | **mT1 (t)** | **d(NS423 x T1, n)** | **d(NS423 x T1, t)** | **Mid-parent value (m)** | **mNS423 (n)** | **mT1 (t)** | **d(NS423 x T1, n)** | **d(NS423 x T1, t)** | **mNS423 (n)** | | **mT1 (t)** | | **mNS423 (n)** | | **mT1 (t)** | |
| 0.18 | 0.71 | 0.11 | 0.34 | 0.45 | -0.27 | 0.27 | -0.34 | -0.11 | 1.28 | 0.41 | -1.28 | -0.41 | OD | PD | **UD** | **PD** |
|  |  |  |  |  |  |  |  |  | **d(NS426 x T1, n)** | **d(NS426 x T1, t)** | **d(NS426 x T1, n)** | **d(NS426 x T1, t)** | **d(NS426 x T1, n)** | **d(NS426 x T1, t)** | **d(NS426 x T1, n)** | **d(NS426 x T1, t)** |
| **mNS426 (n)** | **mT1 (t)** | **d(NS426 x T1, n)** | **d(NS426 x T1, t)** | **Mid-parent value (m)** | **mNS426 (n)** | **mT1 (t)** | **d(NS426 x T1, n)** | **d(NS426 x T1, t)** | **mNS426 (n)** | | **mT1 (t)** | | **mNS426 (n)** | | **mT1 (t)** | |
| 0.08 | 0.71 | 0.06 | 0.09 | 0.40 | -0.32 | 0.32 | -0.34 | -0.31 | 1.07 | 0.98 | -1.07 | -0.98 | D | D | D | D |

Light blue: values represent the average of three replicates of cat relative gene expression in the parental strains.

Light red: values represent the average of three replicates of *cat* relative gene expression of the hybrid strains.

(n) is the mitochondrial type inherited from the dN001 (n) strain.

(t) is the mitochondrial type inherited from the dT009 (t) strain.

(m), (a), (d) and (d/a) were calculated as it was described in Materials and methods.

C: Codominance; PD: Partial Dominance; D: Full Dominance; OD: Overdominance; UD: Underdominance

**Table S8**. **MI8.** **M**ode of **I**nheritance (**MI**) of the *gpx* gene in fast- and slow-growing hybrids.

| **Relative expression of *gpx*** | | | | | **Additive genetic deviation (a)** | | **Dominant genetic deviation (d)** | | **d/a** | | | | **Mode of inheritance** | | | |
| --- | --- | --- | --- | --- | --- | --- | --- | --- | --- | --- | --- | --- | --- | --- | --- | --- |
| **Parental strains** | | **Hybrid strains** | |  |  |  |  |  | **d(NF418 x T1, n)** | **d(NF418 x T1, t)** | **d(NF418 x T1, n)** | **d(NF418 x T1, t)** | **d(NF418 x T1, n)** | **d(NF418 x T1, t)** | **d(NF418 x T1, n)** | **d(NF418 x T1, t)** |
| **mNF418 (n)** | **mT1 (t)** | **d(NF418 x T1, n)** | **d(NF418 x T1, t)** | **Mid-parent value (m)** | **mNF418 (n)** | **mT1 (t)** | **d(NF418 x T1, n)** | **d(NF418 x T1, t)** | **mNF418 (n)** | | **mT1 (t)** | | **mNF418 (n)** | | **mT1 (t)** | |
| 0.08 | 0.12 | 0.15 | 0.07 | 0.10 | -0.02 | 0.02 | 0.05 | -0.03 | -2.38 | 1.62 | 2.38 | -1.62 | UD | OD | **OD** | **UD** |
|  |  |  |  |  |  |  |  |  | **d(NF419 x T1, n)** | **d(NF419 x T1, t)** | **d(NF419 x T1, n)** | **d(NF419 x T1, t)** | **d(NF419 x T1, n)** | **d(NF419 x T1, t)** | **d(NF419 x T1, n)** | **d(NF419 x T1, t)** |
| **mNF419 (n)** | **mT1 (t)** | **d(NF419 x T1, n)** | **d(NF419 x T1, t)** | **Mid-parent value (m)** | **mNF419 (n)** | **mT1 (t)** | **d(NF419 x T1, n)** | **d(NF419 x T1, t)** | **mNF419 (n)** | | **mT1 (t)** | | **mNF419 (n)** | | **mT1 (t)** | |
| 0.08 | 0.12 | 0.07 | 0.08 | 0.10 | -0.02 | 0.02 | -0.03 | -0.02 | 1.39 | 0.95 | -1.39 | -0.95 | OD | D | UD | D |
|  |  |  |  |  |  |  |  |  | **d(NS423 x T1, n)** | **d(NS423 x T1, t)** | **d(NS423 x T1, n)** | **d(NS423 x T1, t)** | **d(NS423 x T1, n)** | **d(NS423 x T1, t)** | **d(NS423 x T1, n)** | **d(NS423 x T1, t)** |
| **mNS423 (n)** | **mT1 (t)** | **d(NS423 x T1, n)** | **d(NS423 x T1, t)** | **Mid-parent value (m)** | **mNS423 (n)** | **mT1 (t)** | **d(NS423 x T1, n)** | **d(NS423 x T1, t)** | **mNS423 (n)** | | **mT1 (t)** | | **mNS423 (n)** | | **mT1 (t)** | |
| 0.19 | 0.12 | 0.43 | 0.33 | 0.16 | 0.04 | -0.04 | 0.27 | 0.17 | 7.66 | 4.80 | -7.66 | -4.80 | **OD** | **OD** | UD | UD |
|  |  |  |  |  |  |  |  |  | **d(NS426 x T1, n)** | **d(NS426 x T1, t)** | **d(NS426 x T1, n)** | **d(NS426 x T1, t)** | **d(NS426 x T1, n)** | **d(NS426 x T1, t)** | **d(NS426 x T1, n)** | **d(NS426 x T1, t)** |
| **mNS426 (n)** | **mT1 (t)** | **d(NS426 x T1, n)** | **d(NS426 x T1, t)** | **Mid-parent value (m)** | **mNS426 (n)** | **mT1 (t)** | **d(NS426 x T1, n)** | **d(NS426 x T1, t)** | **mNS426 (n)** | | **mT1 (t)** | | **mNS426 (n)** | | **mT1 (t)** | |
| 0.25 | 0.12 | 0.29 | 0.24 | 0.19 | 0.06 | -0.06 | 0.10 | 0.05 | 1.59 | 0.84 | -1.59 | -0.84 | OD | PD | UD | PD |

Light blue: values represent the average of three replicates of gpx relative gene expression in the parental strains.

Light red: values represent the average of three replicates of *gpx* relative gene expression of the hybrid strains.

(n) is the mitochondrial type inherited from the dN001 (n) strain.

(t) is the mitochondrial type inherited from the dT009 (t) strain.

(m), (a), (d) and (d/a) were calculated as it was described in Materials and methods.

C: Codominance; PD: Partial Dominance; D: Full Dominance; OD: Overdominance; UD: Underdominance
